# Longitudinal evaluation of proton magnetic resonance spectroscopy metabolites as biomarkers in Huntington’s disease

**DOI:** 10.1093/braincomms/fcac258

**Published:** 2022-10-12

**Authors:** Alexander J Lowe, Filipe B Rodrigues, Marzena Arridge, Enrico De Vita, Eileanoir B Johnson, Rachael I Scahill, Lauren M Byrne, Rosanna Tortelli, Amanda Heslegrave, Henrik Zetterberg, Edward J Wild

**Affiliations:** UCL Huntington’s Disease Centre, UCL Queen Square Institute of Neurology, University College London, London WC1N 3BG, UK; UCL Huntington’s Disease Centre, UCL Queen Square Institute of Neurology, University College London, London WC1N 3BG, UK; Lysholm Department of Neuroradiology, National Hospital for Neurology and Neurosurgery, London WC1N 3BG, UK; Lysholm Department of Neuroradiology, National Hospital for Neurology and Neurosurgery, London WC1N 3BG, UK; Department of Radiology, Great Ormond Street Hospital, London WC1N 3JH, UK; Department of Biomedical Engineering, School of Biomedical Engineering & Imaging Sciences, King’s College London, King’s Health Partners, St Thomas’ Hospital, London SE1 7EH, UK; UCL Huntington’s Disease Centre, UCL Queen Square Institute of Neurology, University College London, London WC1N 3BG, UK; UCL Huntington’s Disease Centre, UCL Queen Square Institute of Neurology, University College London, London WC1N 3BG, UK; UCL Huntington’s Disease Centre, UCL Queen Square Institute of Neurology, University College London, London WC1N 3BG, UK; UCL Huntington’s Disease Centre, UCL Queen Square Institute of Neurology, University College London, London WC1N 3BG, UK; UK Dementia Research Institute at University College London, Fluid Biomarker Laboratory, London WC1E 6BT, UK; Department of Neurodegenerative Disease, UCL Queen Square Institute of Neurology, University College London, London WC1N 3BG, UK; UK Dementia Research Institute at University College London, Fluid Biomarker Laboratory, London WC1E 6BT, UK; Department of Neurodegenerative Disease, UCL Queen Square Institute of Neurology, University College London, London WC1N 3BG, UK; Department of Psychiatry and Neurochemistry, Institute of Neuroscience and Physiology, the Sahlgrenska Academy at the University of Gothenburg, Mölndal SE-413 45, Sweden; Clinical Neurochemistry Laboratory, Sahlgrenska University Hospital, Mölndal SE-413 45, Sweden; Hong Kong Center for Neurodegenerative Diseases, Fluid Biomarker Laboratory, 17 Science and Technology W Ave, Science Park, Hong Kong, China; UCL Huntington’s Disease Centre, UCL Queen Square Institute of Neurology, University College London, London WC1N 3BG, UK

**Keywords:** biomarkers, Huntington’s disease, magnetic resonance spectroscopy, cerebrospinal fluid

## Abstract

Proton magnetic resonance spectroscopy is a non-invasive method of exploring cerebral metabolism. In Huntington’s disease, altered proton magnetic resonance spectroscopy-determined concentrations of several metabolites have been described; however, findings are often discrepant and longitudinal studies are lacking. Proton magnetic resonance spectroscopy metabolites may represent a source of biomarkers, thus their relationship with established markers of disease progression require further exploration to assess prognostic value and elucidate pathways associated with neurodegeneration. In a prospective single-site controlled cohort study with standardized collection of CSF, blood, phenotypic and volumetric imaging data, we used 3 T proton magnetic resonance spectroscopy in conjunction with the linear combination of model spectra method to quantify seven metabolites (total *n*-acetylaspartate, total creatine, total choline, myo-inositol, GABA, glutamate and glutathione) in the putamen of 59 participants at baseline (15 healthy controls, 15 premanifest and 29 manifest Huntington’s disease gene expansion carriers) and 48 participants at 2-year follow-up (12 healthy controls, 13 premanifest and 23 manifest Huntington’s disease gene expansion carriers). Intergroup differences in concentration and associations with CSF and plasma biomarkers; including neurofilament light chain and mutant Huntingtin, volumetric imaging markers; namely whole brain, caudate, grey matter and white matter volume, measures of disease progression and cognitive decline, were assessed cross-sectionally using generalized linear models and partial correlation. We report no significant groupwise differences in metabolite concentration at baseline but found total creatine and total *n*-acetylaspartate to be significantly reduced in manifest compared with premanifest participants at follow-up. Additionally, total creatine and myo-inositol displayed significant associations with reduced caudate volume across both time points in gene expansion carriers. Although relationships were observed between proton magnetic resonance spectroscopy metabolites and biofluid measures, these were not consistent across time points. To further assess prognostic value, we examined whether baseline proton magnetic resonance spectroscopy values, or rate of change, predicted subsequent change in established measures of disease progression. Several associations were found but were inconsistent across known indicators of disease progression. Finally, longitudinal mixed-effects models revealed glutamine + glutamate to display a slow linear decrease over time in gene expansion carriers. Altogether, our findings show some evidence of reduced total *n*-acetylaspartate and total creatine as the disease progresses and cross-sectional associations between select metabolites, namely total creatine and myo-inositol, and markers of disease progression, potentially highlighting the proposed roles of neuroinflammation and metabolic dysfunction in disease pathogenesis. However, the absence of consistent group differences, inconsistency between baseline and follow-up, and lack of clear longitudinal change suggests that proton magnetic resonance spectroscopy metabolites have limited potential as Huntington’s disease biomarkers.

## Teaser

The study by Lowe et al. uses magnetic resonance spectroscopy to investigate the biomarker potential of neurochemical metabolites in Huntington’s disease. Cross-sectional associations were observed between metabolites and prognostic measures; however, the absence of consistent group differences and lack of clear longitudinal change indicates limited biomarker potential in Huntington’s disease.

## Graphical Abstract

**Graphical abstract:**
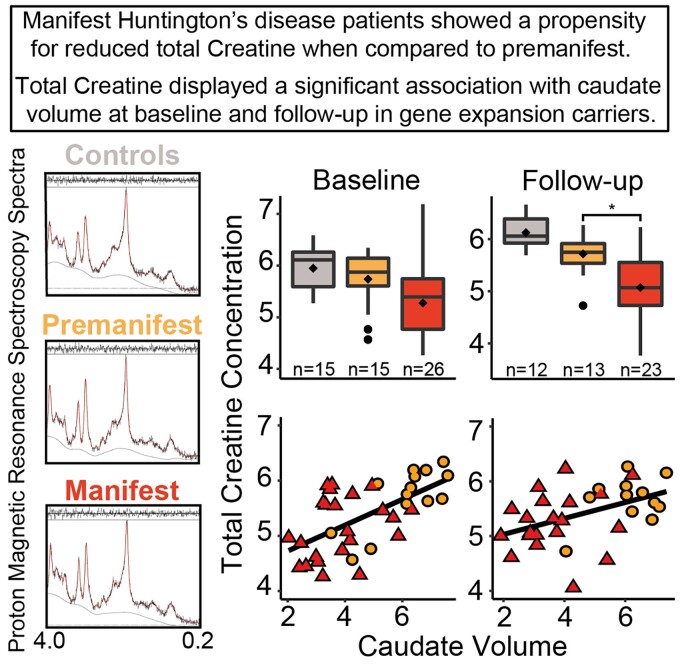

## Introduction

Huntington’s disease is a neurodegenerative disease characterized by progressive motor, psychiatric and cognitive dysfunction.^1^ Invariably fatal, Huntington’s disease is caused by an autosomal dominant mutation in the *HTT* gene, producing a CAG repeat expansion in the ubiquitously expressed huntingtin protein (HTT).^2^ This mutated pathogenic product (mHTT) causes a wide array of toxicities and disruption of downstream pathways, resulting in neuronal death.^3^ With genetic testing, the development of Huntington’s disease can be accurately predicted; however, there remains a need to discover clinically relevant biomarkers with the ability to detect and quantify pathogenic change, pharmacological target engagement and treatment response.^4^ Due to its non-invasive nature, accessibility and the potential to standardise parameters across multiple sites, neuroimaging is a valuable source of information about progression and prognosis^4^ and has been utilized in Huntington’s disease to explore cross-sectional and longitudinal changes in brain structure, metabolism and activation patterns.^5–12^

Proton magnetic resonance spectroscopy (^1^H-MRS) is a non-invasive method of exploring cerebral metabolism, and represents an interesting avenue in biomarker research as neurometabolic alterations may occur prior to the emergence of structural and functional change.^13, 14^ The number of quantifiable metabolites depends on several factors including pulse sequence, spectral resolution and signal-to-noise ratio (SNR),^15^ all of which can be influenced by the magnetic field strength, with higher strengths providing increased sensitivity and spectral resolution.^16, 17^ In the context of neurodegenerative disease, tNAA (*n*-acetylaspartate + *n*-acetylaspartate-glutamate), tCho (phosphocholine + glycophosphocholine), tCre (creatine + phosphocreatine) and myo-inositol (MI) are considered respective biomarkers for neuro-axonal viability and mitochondrial dysfunction,^18, 19^ cellular proliferation and neuronal membrane turnover,^20, 21^ brain energy metabolism and gliosis^22^ and astrocytic density.^23^ Due to its relative stability in pathological conditions, creatine (Cr) is often used as an internal reference,^24^ however, it is affected in Huntington’s disease, so ^1^H-MRS metabolites may be normalized to unsuppressed water signal, allowing the accurate identification of biochemical change in the brain.^25^

In Huntington’s disease, altered concentrations of several ^1^H-MRS metabolites have been described in both premanifest (PreHD) and manifest gene expansion carriers (HD) across multiple brain regions;^26–33^ however, other studies have reported no significant differences in metabolite concentrations when comparing patient cohorts to healthy controls (CTR) (Table 1).^34, 35^ These discrepant findings are likely due, in part, to sample size variations, patient heterogeneity and differences in spatial/spectral resolution. Recent work leveraging 7-tesla MRI^35^ found lower metabolite levels to correspond to poorer clinical, cognitive and behavioural scores, similar to work leveraging the TRACK-HD cohort in which tNAA displayed a significant negative correlation with disease burden score (DBS) across PreHD and early HD, further demonstrating its role as a marker of clinical decline.^25^ Longitudinal analyses have produced mixed results thus far, with reduced tNAA and Cho in the putamen, and Cr and MI in the caudate, reported,^36^ whereas other have reported no longitudinal change in metabolite concentration.^36, 37^ Importantly, the latter two studies normalized metabolite values to unsuppressed water signal, whilst also benefitting from high SNR and large sample sizes; however, the role of ^1^H-MRS metabolites as prognostic biomarkers remains debatable and warrants further study.

**Table 1:** Summary of ^1^H-MRS studies in Huntington’s disease

Authors	Group comparison	Brain region	Metabolic changes in gene expansion carriers
Sturrock *et al.*^25^	HD versus CTR	Putamen	↓ tNAA, ↓ NAA, ↓ tCre, ↓ Glu, ↑ MI, ↑ tCho
	HD versus PreHD	Putamen	↓ tNAA, ↓ NAA, ↓ tCre, ↓ Glu, ↑ MI, ↑ tCho
	PreHD versus CTR	Putamen	↓ NAA
Gomez-Anson *et al.*^26^	PreHD versus CTR	Frontal cortex	↓ Cho
		Basal ganglia	No change
Ruocco *et al.*^27^	HD versus CTR	Thalamus	↓ NAA/Cr
Sanchez-Pernaute *et al.*^28^	PreHD/HD versus CTR	Basal ganglia	↓ NAA, ↓ Cre
Hoang *et al.*^29^	HD versus CTR	Occipital cortex	↓ NAA/Cr
		Putamen	↓ NAA/Cr, ↓ Cre, ↑ MI, ↑Cho/Cr
Adanyeguh *et al.*^30^	HD versus CTR	Visual cortex	↑ tCre,
		Striatum	↓ Glu, ↓ tCre
Jenkins *et al.*^31^	HD versus CTR	Striatum	↓ tNAA/Cr, ↑ Cho/Cr, ↑ Lac
		Occipital cortex	↑ Lac
Jenkins *et al.*^32^	HD versus CTR	Striatum	↓ tNAA/Cr, ↑ Cho/Cr, ↑ Lac
		Occipital cortex	↑ Cho/Cr, ↑ Lac
Clarke *et al.*^33^	HD versus CTR	Striatum	↓ NAA, ↓ Cre
Van Oostrum *et al.*^34^	PreHD versus CTR	Putamen	No change
Van den Bogaard *et al.*^35^	HD versus CTR	Hypothalamus	No change
		Thalamus	No change
		Caudate	↓ NAA, ↓ Cre
		Putamen	↓ NAA, ↓ Cre, ↓ GLX
		Prefrontal cortex	No change
	PreHD versus CTR	Hypothalamus	No change
		Thalamus	No change
		Caudate	No change
		Putamen	No change
		Prefrontal cortex	No change

CTR, healthy controls; PreHD, premanifest gene expansion carriers; HD, manifest gene expansion carriers; tNAA, total *n*-acetylaspartate; Cho, choline; Cr, creatine; tCr, total creatine; tCho, total choline Glu, glutamate; GLX, glutamine and glutamate; MI, myo-inositol; Lac, lactate; ↓, reduced concentration; ↑, increased concentration.

The relationship between biofluid markers and ^1^H-MRS metabolites requires further exploration, as combining direct and non-invasive quantifications of biochemical alterations could improve the value of both biomarker modalities. The concentration of neurofilament light chain (NfL), measured in CSF and blood, represents axonal damage and is a prognostic biomarker of neurodegeneration.^38–40^ Its relationship with ^1^H-MRS metabolites, particularly tNAA, MI and tCre, has not previously been examined in Huntington’s disease to our knowledge. In patients with HIV, elevated levels of CSF NfL have been shown to correlate with decreased NAA/Cre in multiple brain regions, indicating compromised neuronal health and stability.^41^ In multiple sclerosis patients, the same inverse relationship has been observed between serum NfL and NAA/Cre at baseline, yet is not present at 12 and 36 months following haematopoietic stem cell transplantation.^42^ Given the elevated concentration of NfL in Huntington’s disease, we hypothesized that an inverse relationship with tNAA and tCre would be present in the putamen of Huntington’s disease patients. Furthermore, mHTT can be accurately quantified in CSF following its release from damaged neurons^43, 44^ and displays strong associations with CSF NfL.^39, 40, 44^ As such, we would expect to observe the same relationships with CSF mHTT. In Alzheimer’s disease, reduced NAA/Cre and increased MI/Cre have been associated with increased *P*- and t-tau, and decreased CSF amyloid-beta (Aβ42), across several brain regions.^45–47^ The association between MI and tau, another established marker of neurodegeneration,^48^ is thought to be driven by activation of MI-rich astrocytes and microglia.^46^ Additionally, given that neuroinflammation represents a key pathogenic component,^49^ and source of CSF biomarkers,^50^ in Huntington’s disease, we expect to observe positive correlations between MI and all biofluid markers, reflecting the contribution of excessive neuroinflammatory response on disease pathogenesis.

Using the HD-CSF cohort,^39, 40^ a large prospective sample of gene expansion carriers and matched CTRs with CSF and blood plasma collection and 3 T MRI acquisition, we employed ^1^H-MRS to conduct a cross-sectional and longitudinal neurochemical analysis in the putamen. We aimed to establish if significant metabolic alterations are present in the putamen of Huntington’s disease patients, and if significant associations exist between metabolite concentration and measures of clinical progression, including composite Unified Huntington’s Disease Rating Scale (cUHDRS) and DBS score, cognitive decline, prognostic biomarkers quantified in CSF and blood and volumetric MRI measurements.

## Materials and methods

### Participants

HD-CSF was a prospective single-site study with standardized longitudinal collection of CSF, blood and phenotypic data (online protocol: 10.5522/04/11828448.v1) from HD, PreHD and CTRs. Eighty participants were recruited (20 CTR, 20 PreHD and 40 HD) based on a priori sample size calculations to detect cross-sectional and longitudinal differences in CSF mHTT between CTRs and gene expansion carriers.^44^ 3 T MRI scans were optional for HD-CSF participants. The present study used ^1^H-MRS data obtained from 59 participants at baseline and 48 at longitudinal follow-up after 24 months. A total of 42 participants had data available at both time points. Manifest Huntington’s disease was defined as Unified Huntington’s Disease Rating Scale (UHDRS)^51^ diagnostic confidence level (DCL) = 4 and CAG repeat length > 36. PreHD had CAG repeat length > 40 and DCL < 4. CTR were contemporaneously recruited, drawn from a population with a similar age to patients, and clinically well, so the risk of incidental neurological diseases was very low. Consent, inclusion and exclusion criteria, clinical assessment, CSF collection and storage were as previously described.^39, 52^ Baseline and longitudinal 24-month follow-up samples from HD-CSF have been used for this study.

### Ethical approval

Ethical approval was given by the London Camberwell St Giles Research Ethics Committee (15/LO/1917), with all participants providing written informed consent prior to enrolment. This study was performed in accordance with the principles of the Declaration of Helsinki, and the International Conference on Harmonization Good Clinical Practice standards.

### Clinical assessments

Relevant aspects of clinical phenotype were quantified using the UHDRS.^51^ A composite UHDRS (cUHDRS) score was generated for each subject to provide a single measure of motor, cognitive and global functioning decline. This composite score is computed using the following formula (total functional capacity, TFC; Total Motor Score, TMS; Symbol Digit Modality Test, SDMT; Stroop Word Reading, SWR):

$$\left[\begin{array}{c} \left(\frac{\mathrm{TFC}-10.4}{1.9}\right)-\left(\frac{\mathrm{TMS}-29.7}{14.9}\right)\;\\ +\left(\frac{\mathrm{SDMT}-28.4}{11.3}\right)+\left(\frac{\mathrm{SWR}-66.1}{20.1}\right) \end{array}\right]+10$$

cUHDRS score has been found to display the strongest relationship to Huntington’s disease brain pathology and enhanced sensitivity to clinical change in early manifest disease.^53^ DBS was calculated for each gene expansion carrier using the formula [CAG repeat length—35.5] × age.^54^ DBS estimates cumulative pathology exposure as a function of CAG repeat length and the time exposed to the effects of the expansion and has been shown to predict several features of disease progression including striatal pathology.^11, 54^

### Volumetric MRI acquisition

T_1_-weighted MRI data were acquired on a 3 T Siemens Prisma scanner using a protocol optimized for this study. Images were acquired using a 3D magnetization-prepared 180 degrees radio-frequency pulses and rapid gradient-echo (MPRAGE) sequence with a repetition time (TR) = 2000 ms, echo time (TE) = 2.05 ms, inversion time = 850 ms, flip angle of 8 degrees and matrix size = 256 × 240 mm. Two hundred and fifty-six coronal partitions were collected to cover the entire brain with a slice thickness of 1.0 mm. Parallel imaging acceleration [GeneRalized Autocalibrating Partial Parallel Acquisition (GRAPPA), acceleration factor (R) = 2] was used and 3D distortion correction was applied to all images. Global (whole brain, grey matter and white matter) and regional (total caudate volume) volumetric measures were computed using the previously described methodology^39, 40, 55^ and adjusted for total intracranial volume (TIV). In brief, volumetric regions of the whole brain were generated using Medical Image Display Analysis Software (MIDAS),^56^ total caudate volume (henceforth ‘caudate volume’) was generated using MALP-EM^57^ and grey/white matter volume was measured via voxel-based morphometry.^58^ Changes in whole brain and caudate volume were calculated via the boundary shift integral method,^57, 59^ whereas a fluid-registration approach^60^ was applied to calculate grey and white matter change. Follow-up volumes were computed by subtracting atrophy amount from baseline volumes. Putamen volume was not quantified as part of HD-CSF, as it is challenging to quantify reliably and does not perform as well as the caudate as a longitudinal measure of disease progression.^11^ As such, the association between ^1^H-MRS metabolites and atrophy within this structure was not explored in this study.

### Magnetic resonance spectroscopy and LCModel quantification

**Figure 1:**
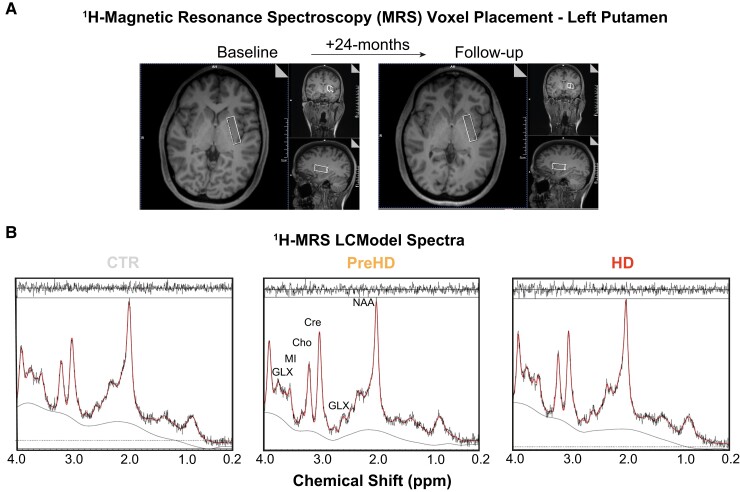
**^1^H-MRS voxel placement and LCModel spectra**: (**A**) Example voxel placement in left putamen displayed at baseline and follow-up. For the voxel placement in the second ^1^H-MRS acquisition, the images from the first scanning were used to match the anatomical location. (**B**) LCModel output generated from healthy controls (CTR), premanifest (PreHD) and manifest (HD) gene expansion carriers, with black and red lines representing the raw spectrum and model fit overlaid on raw data, respectively. Output peaks represent specific metabolite concentrations. Several metabolites have been labelled on the PreHD spectra for illustrative purposes. ppm, parts per million.

All scans were performed using 3 T Siemen’s scanner (Prisma VE11C) with 64 channels RF head coil. For spectroscopy, the manufacturer’s single voxel PRESS sequence was used with the following parameters: TE = 30 ms; TR = 2000 ms; vector size (number of points in the time domain) = 2048; spectral width = 2400 Hz. Spectra was acquired from a rectangular volume of interest (VOI) located in the left putamen: 35 × 10 × 15 mm3 (Fig. 1). Adjustments included the following: transmitter gain, receiver gain, shimming (3D Gradient-Echo followed by manual adjustments to achieve an average water linewidth of 9.73 (0.98) Hz), and water suppression. Water suppressed spectrum was acquired with 160 averages with a reference scan (unsuppressed spectrum 4 averages).

LCModel (v6.3-1L) spectra of 18 metabolites were included in the basis data set together with. simulated^61^ model spectra for macromolecules at the following positions: 0.91, 1.21, 1.43, 1.67, 1.95, 2.08, 2.25 and 3 ppm and lipids at 0.9, 1.3 and 2.0 ppm, complied with the background fitted with spline functions. Metabolite levels were estimated using internal water as a reference. Of the 18 metabolites measures, we selected seven to be included in the main analysis: tNAA, tCre, tCho, MI, glutathione (GSH), GABA and Glutamate + Glutamine (GLX). This decision was based on a review of the literature and potential biological relevance to Huntington’s disease and neurodegeneration. The LCModel produces standard deviations (%SDs) for each metabolite as a measurement of reliability, with SDs below 20% considered reliable. Only GABA had a mean %SD of >20% at both baseline and follow-up. As a quality control measure, we removed any subject with a %SD ≥ 100 from the analyses. This was applied to all metabolites, resulting in the removal of subjects from the GABA cohort only (eight from baseline and six from follow-up; Supplementary Table 1). Spectra with LCM-reported SNR (defined as the ratio of the maximum in the spectrum-minus-Baseline over the Analysis Window to twice the rms Residuals) < 6 were deemed unacceptable for further analysis due to the presence of artefacts, and/or inaccurate fitting of spectra, and excluded. Correction for CSF partial volume effect (PVE) was completed within spectroscopy voxels using the following method: T_1_-weighted 3D volumes were segmented to provide partial volume maps for grey matter, white matter, and CSF. Using the location and orientation parameters obtained from Siemens.rda files, ^1^H-MRS voxels were co-registered onto the T_1_ 3D volume to generate a voxel mask. These masks were then overlaid on the segmented 3D T_1_, and a CSF PVE value was computed for everyone by dividing 1/VOI tissue fraction. The latter of which was obtained using the following formula:

$$\frac{Single\;voxel\;spectroscopy\;(SVS)\;voxel\;size\;masked\;by\;grey\;matter+SVS\;voxel\;size\;masked\;by\;white\;matter}{SVS\;voxel\;size}$$

### Participant characteristics

At baseline, our cohort consisted of 15 CTRs and 44 Huntington’s gene expansion carriers, of whom 15 were classified as PreHD and 29 as HD. Three HD participants had an SNR value of <6 and were excluded from subsequent analysis, resulting in a final baseline sample size of 56. Groups were equally matched for gender (χ^2^ = 0.002, *P* = 0.99) and CAG repeat length (among gene expansion carriers) but as expected, displayed significant differences in clinical, cognitive, volumetric and biofluid measures (Supplementary Table 2). A significant difference in age was observed with HD being significantly older than PreHD participants due to being more advanced in their disease course.

Our follow-up cohort was smaller, consisting of 12 CTRs and 36 gene expansion carriers (13 PreHD and 23 HD), but largely similar in terms of demographics. No subjects were removed due to poor SNR value. Relationships that were significant in the baseline sample were also significant in at follow-up (Supplementary Table 2).

When analysing GABA, eight subjects were removed at baseline and six at follow-up due to high %SD values (≥100), resulting in a smaller sample size (*n* = 48 at baseline and 42 at follow-up) for this metabolite.

### Biofluid collection and processing

CSF and matched plasma were obtained as previously described.^39, 40^ All collections were standardized for time of day after overnight fasting and processed within 30 min of collection using standardized equipment. Blood was collected within 10 min of CSF and processed to plasma. Biosamples were frozen and stored at −80°C.

### Analyte quantification

Analytes were quantified as previously described.^39, 40^

CSF and plasma NfL were quantified in duplicate using the Neurology 4-Plex B assay on the Simoa HD-1 Analyzer (Quanterix). The limit of detection was 0.105 pg/mL, and lower limit of quantification was 0.500 pg/mL. NfL was above the lower limit of quantification in all samples. The intra-assay coefficients of variation (CV) (calculated as the mean of the CVs for each sample’s duplicate measurements) for CSF NfL and plasma NfL were 5.0 and 3.7%, respectively. The inter-assay CVs (calculated as the mean of the CVs for analogous spiked positive controls provided by the manufacturer and used in each well plate) for CSF NfL and plasma NfL were 2.7 and 8.4%, respectively. CSF and plasma tau was quantified in duplicate using the same assay and analyser as NfL, with an limit of detection of 0.041 pg/mL and lower limit of quantification of 0.125 pg/mL. The inter- and intra-assay CV for plasma and CSF tau were 2.0 and 3.9 and 4.9 and 6.5%, respectively. CSF mHTT was quantified in triplicate using the 2B7-MW1 immunoassay (SMC Erenna platform, Merck). The limit of detection was 8 fM, lower limit of quantification was 25 fM and the intra-assay CV was 14.1%.

All biofluid measures, except CSF mHTT, were log-transformed to meet model assumptions.

### Statistical analysis

Statistical analysis was performed with Stata IC 15 software (StataCorp, TX, USA). The distributions of all metabolite concentrations were visually assessed using kernel density estimate plots and Q-Q plots. Data transformations were not required to meet model assumptions (Supplementary Fig. 1). Differences in demographic (age, gender and CAG repeat length), clinical (cUHDRS, DBS, TFC and TMS), cognitive (SDMT, SWR, Verbal Fluency Categorical, VFC; and Stroop Colour Naming, SCN), volumetric (Whole brain, caudate, grey and white matter volume) and biofluid (CSF NfL, CSF mHTT, CSF Tau, Plasma NfL and Plasma Tau) measures were examined using Chi squared tests and generalized linear models. Models were not adjusted for covariates at this stage.

To reduce the risk of Type 1 error, we preselected tNAA, tCre, tCho and MI as primary outcome measures based on the published ^1^H-MRS literature in Huntington’s disease (see introduction) and protocol design. GSH, GABA and GLX were designated as secondary outcome measures as the protocol was not specifically optimized for their quantification; however, they still possess biological relevance in the context of neurodegenerative disease. Our decision to divide the metabolites into ‘Primary’ and ‘Secondary’ in no way reflects the biological importance of the metabolites. Age and gender were considered potentially confounding variables, thus their relationship with metabolite concentration was examined using Pearson’s correlation and independent samples *t*-tests.

To investigate group differences, we applied generalized linear regression models with metabolite concentration as the dependant variable. When comparing CTRs to PreHD, age and CSF PVE were included in the model as independent variables. To assess associations beyond the combined effect of age and CAG repeat count, models including gene expansion carriers only (i.e. PreHD versus HD) were run with CAG repeat count included as an additional covariate. We treat Huntington’s disease as a biological continuum, thus other comparisons i.e. CTR versus HD, were not undertaken. Additionally, we applied an inverse weighting to the %SD values of each metabolite to compensate for any variations in LCModel output quality. By including both age and CAG as covariates, accurate assessments of associations can be made, independent of known predictors. Due to the exploratory nature of the study, tests were not adjusted for multiple comparisons.

Associations between metabolites and clinical, cognitive, volumetric measures and established biofluid markers were explored cross-sectionally using Pearson’s partial correlation controlling for age and CSF PVE, then additionally controlling for CAG repeat count. For all correlation analyses, we combined PreHD and HD into a single group, henceforth called Huntington’s disease mutation carriers (HDMCs). Associations in the CTR group were not explored in this study. DBS is a product of age and CAG, as such, we did not adjust for these variables when analysing DBS. In keeping with our regression analysis, we removed any subject with a %SD ≥ 100 and applied inverse weighting to the %SD values of the remaining subjects. This process was applied to each metabolite individually. Additionally, we performed unweighted, bootstrapped (1000 repetitions) partial correlations in which bias-corrected and accelerated 95% confidence intervals (95% CI) were calculated for correlation coefficients. Metabolites were deemed to have prognostic potential if a significant relationship was observed across all four correlation models [Inverse weighting controlling for age and CSF PVE (i), then additionally controlling for CAG repeat length (ii), followed by bootstrapping controlling for age and CSF PVE (iii), and then additionally controlling for CAG repeat length (iv)]. Although stringent, we chose this method to allow identification of ^1^H-MRS metabolites that demonstrate the strongest biomarker potential. Unless otherwise stated, *P*-values and correlation coefficients shown in text and figure legends are obtained from Pearson’s partial correlation controlling for age, CSF PVE and CAG repeat length, and bootstrapped with 1000 repetitions. No adjustments were made for multiplicity.

The cross-sectional statistical analyses outlined above was also applied to the follow-up dataset. We reasoned that the 2 years’ disease progression in all Huntington’s disease patients might outweigh the loss of power from participant dropout.

Rate of change for each ^1^H-MRS metabolite and clinical, cognitive and volumetric measures were computed by subtracting baseline from follow-up values and dividing by the time between visits in years. Intergroup differences and correlations were examined using the methods outlined above. Only those subjects with data at both baseline and follow-up were included in this analysis.

To study longitudinal trajectories of the metabolites, we used mixed-effects models with age and CSF PVE as fixed effects, and random effects for participant (intercept) and age (slope), generated independently for CTRs and HDMCs. All available data points were used in this analysis.

### Data availability

The data that support the findings of this study are available on request from the corresponding author, EJW. The data are not publicly available due to their containing information that could compromise the privacy of research participants.

## Results

### ^1^H-MRS test–retest reliability

Test–retest variability, measured via %change across time points, was generally small, ranging from 1.3% for GSH to 16% for GABA when examined over 24 months in 11 CTRs, and from 1.1% for tCho to 6.5% for GLX when two CTRs were re-scanned immediately. Additionally, coefficient of variation values for the two latter CTRs were computed and ranged from 0.8% for tCho and 4.5% for GLX.

### Analysis of metabolite group differences

Baseline analysis in CTRs revealed MI to be significantly associated with age (*r* = 0.64, *P* = 0.01), and tCre and GLX to display significant gender differences (*t* = −2.35, *P* = 0.04; *t* = −2.62, *P* = 0.02, respectively). Therefore, in addition to age, gender was included as a covariate in all subsequent baseline analysis of tCre and GLX in CTRs. At follow-up, no significant relationships were observed, thus gender was not controlled for (Supplementary Table 3).

**Figure 2:**
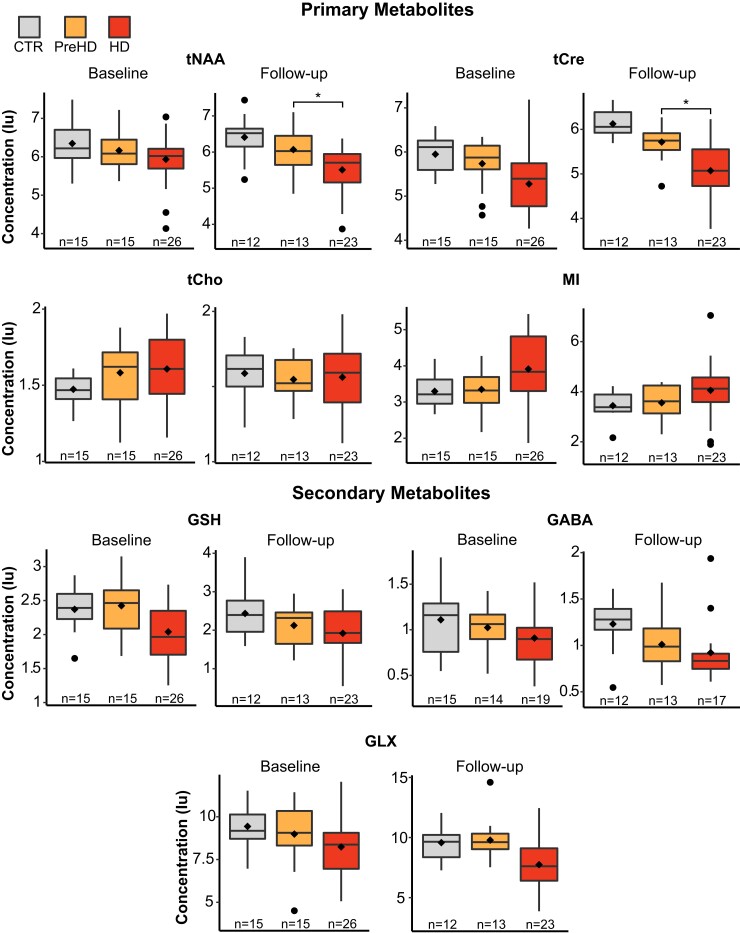
**Group differences in metabolite concentration at baseline and follow-up**. General linear modes controlling for age and CSF PVE revealed statistically significant differences in tNAA (F(4,43) = [3.98], *P* < 0.01) and tCre (F(4,43) = [8.16], *P* < 0.001) concentration at follow-up. HD participants were shown to have reduced concentration compared with PreHD (tNAA, *P* = 0.03, 95% CIs = [−1.20, −0.07]; tCre, *P* < 0.01, 95% CIs = [−1.38, −0.34]). These results remained significant when additionally controlling for CAG repeat length (tNAA, *P* = 0.03, 95% CIs = [−1.40, −0.07]; tCre, *P* = 0.04, 95% CIs = [−1.34, −0.04]). Diamonds represent mean values and ‘*’ represents statistical significance at *P* < 0.05. Tests were not corrected for multiple comparisons. Iu, Institutional units.

At baseline, we found no significant differences in metabolite concentration between groups. Analysis at follow-up revealed tNAA and tCre to display significantly lower concentration in HD, compared with PreHD, when controlling for all covariates (Fig. 2; Table 2).

**Table 2:** Intergroup differences in metabolite concentration at baseline and follow-up

	CTR		PreHD		HD		Model output			
	*n*	Mean (SD)	*n*	Mean (SD)	*n*	Mean (SD)	Adjusted for	Model *P*-value	CTR versus PreHD	PreHD versus HD
**Primary metabolites (Iu)**										
**tNAA**	15	6.35 (0.60)	15	6.16 (0.56)	26	5.93 (0.69)	Age, PVE	0.08	0.75	0.03
							Age, PVE, CAG	N/A	N/A	0.06
Follow-up	12	6.41 (0.61)	13	6.07 (0.60)	23	5.51 (0.67)	Age, PVE	**0.01**	0.29	**0.03**
							Age, PVE, CAG	N/A	N/A	**0.03**
ROC/yr	11	0.10 (0.44)	12	−0.12 (0.41)	19	−0.22 (0.38)	Age, PVE	0.16	0.10	0.51
							Age, PVE, CAG	N/A	N/A	0.23
**tCre**	15	5.95 (0.42)	15	5.74 (0.55)	26	5.28 (0.68)	Age, PVE, Gen	0.01	0.53	0.11
							Age, PVE, CAG	N/A	N/A	0.10
Follow-up	12	6.13 (0.31)	13	5.72 (0.42)	23	5.07 (0.69)	Age, PVE	**<0.001**	0.14	**<0.01**
							Age, PVE, CAG	N/A	N/A	**0.04**
ROC/yr	11	0.18 (0.42)	12	0.01 (0.38)	19	−0.01 (0.36)	Age, PVE, Gen	0.10	0.55	0.30
							Age, PVE, CAG	N/A	N/A	0.22
**tCho**	15	1.47 (0.10)	15	1.58 (0.22)	26	1.61 (0.21)	Age, PVE	0.35	0.15	0.66
							Age, PVE, CAG	N/A	N/A	0.29
Follow-up	12	1.59 (0.18)	13	1.55 (0.16)	23	1.56 (0.25)	Age, PVE	0.12	0.95	0.09
							Age, PVE, CAG	N/A	N/A	0.20
ROC/yr	11	0.09 (0.19)	12	0.02 (0.15)	19	0.02 (0.13)	Age, PVE	0.04	0.69	0.11
							Age, PVE, CAG	N/A	N/A	0.10
**MI**	15	3.30 (0.47)	15	3.35 (0.57)	26	3.91 (0.95)	Age, PVE	0.43	0.78	0.28
							Age, PVE, CAG	N/A	N/A	0.41
Follow-up	12	3.44 (0.54)	13	3.56 (0.64)	23	4.05 (1.12)	Age, PVE	0.80	0.94	0.55
							Age, PVE, CAG	N/A	N/A	0.40
ROC/yr	11	0.13 (0.40)	12	0.17 (0.22)	19	0.10 (0.49)	Age, PVE	0.84	0.88	0.40
							Age, PVE, CAG	N/A	N/A	0.79
**Secondary metabolites (Iu)**										
**GSH**	15	2.37 (0.30)	15	2.42 (0.44)	26	2.04 (0.42)	Age, PVE	0.01	0.85	0.05
							Age, PVE, CAG	N/A	N/A	0.11
Follow-up	12	2.44 (0.65)	13	2.12 (0.53)	23	1.92 (0.61)	Age, PVE	0.02	0.05	0.49
							Age, PVE, CAG	N/A	N/A	0.64
ROC/yr	11	0.04 (0.53)	12	−0.17 (0.39)	19	0.04 (0.38)	Age, PVE	0.19	0.10	0.54
							Age, PVE, CAG	N/A	N/A	0.46
**GABA**	15	1.11 (0.39)	14	1.02 (0.23)	19	0.91 (0.29)	Age, PVE	0.08	0.92	0.22
							Age, PVE, CAG	N/A	N/A	0.21
Follow-up	12	1.23 (0.29)	13	1.01 (0.30)	17	0.92 (0.31)	Age, PVE	0.16	0.13	0.32
							Age, PVE, CAG	N/A	N/A	0.56
ROC/yr	11	0.15 (0.41)	11	0.00 (0.23)	11	−0.02 (0.25)	Age, PVE	0.82	0.53	0.61
							Age, PVE, CAG	N/A	N/A	0.45
**GLX**	15	9.44 (1.21)	15	8.99 (1.87)	26	8.24 (1.69)	Age, PVE, Gen	<0.01	0.09	0.24
							Age, PVE, CAG	N/A	N/A	0.13
Follow-up	12	9.58 (1.51)	13	9.79 (1.73)	23	7.74 (2.16)	Age, PVE	<0.01	0.95	0.07
							Age, PVE, CAG	N/A	N/A	0.32
ROC/yr	11	0.20 (1.10)	12	0.69 (1.55)	19	0.02 (1.06)	Age, PVE, Gen	0.10	0.24	0.03
							Age, PVE, CAG	N/A	N/A	0.02

Differences in metabolite concentration and rate of change across disease stage were assessed using general linear models controlling for effects of age and CSF PVE, and additionally controlling for CAG repeat length. Gender was also controlled for when analysing tCre and GLX at baseline when including CTR. P-values are not corrected for multiple comparisons due to exploratory nature of study. Iu, Institutio­nal units; ROC, rate of change; CTR, healthy controls; PreHD, premanifest gene expansion carriers; HD, manifest gene expansion carriers; Gen, Gender; PVE, partial volume effect; tNAA, total N-acetylaspartate; tCr, total creatine; tCho, total choline; MI, myo-inositol; GSH, Glutathione; GLX, glutamine and glutamate.

### Correlation analysis of metabolites and measures of clinical progression in HDMCs

**Figure 3:**
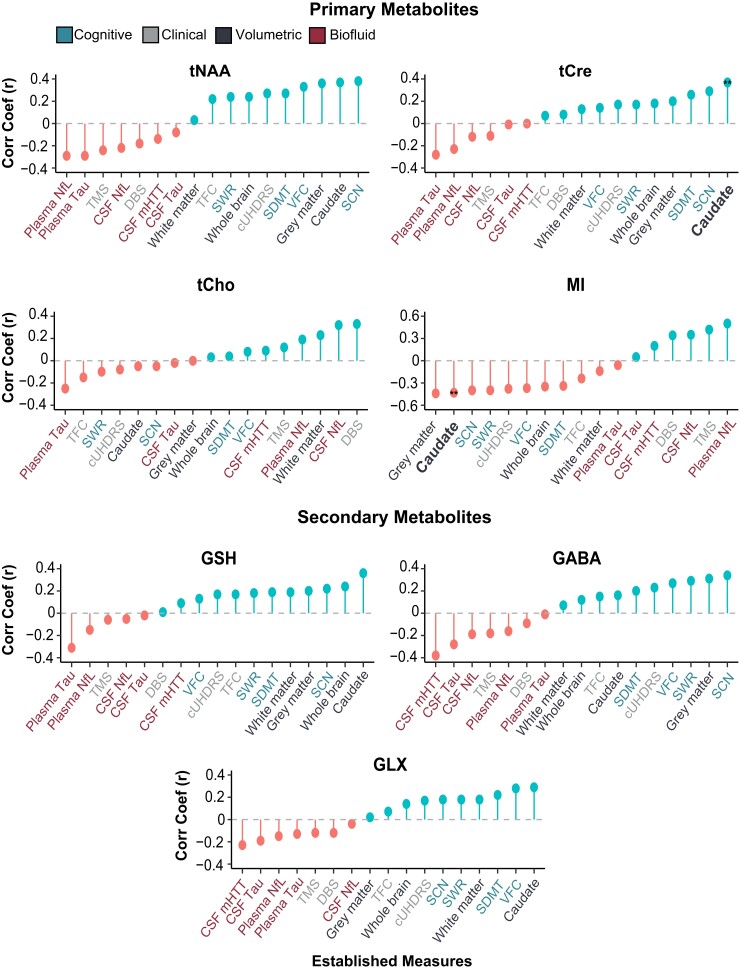
**Associations between metabolites and clinical, cognitive, volumetric and biofluid measures at baseline.** tCre and MI displayed a significant association with caudate volume across all models at baseline (tCre, *r* = 0.45, *P* < 0.01; MI, *r* = −0.41, *P* < 0.01) and follow-up (tCre, *r* = 0.52, *P* < 0.01; MI, *r* = −0.44. *P* < 0.01) in HDMCs. Correlation coefficients displayed in the figure are generated using Pearson’s partial correlation controlling for age and CSF PVE and bootstrapped with 1000 repetitions. Double stars and bold text (**) represent statistical significance (*P* < 0.05) across all correlation models and time points.

At baseline, we found tCho to display a positive association with DBS (*r* = 0.33, *P* < 0.05). When controlling for age and CSF PVE, we found MI to display a negative correlation with cUHDRS and positive correlation with TMS. When additionally controlling for CAG repeat length, these relationships no longer achieved statistical significance (Fig. 3; Supplementary Table 4).

Analysis at follow-up did not replicate any baseline findings; however, we found reduced levels of tCre and GLX to be significantly associated with a reduction in cUHDRS scores (tCre, *r* = 0.40, *P* < 0.01; GLX, *r* = 0.40, *P* < 0.01) and an increase in TMS (tCre, *r* = −0.44, *P* < 0.01; GLX, *r* = −0.47, *P* < 0.01), indicative of a worsening clinical phenotype. These relationships remained significant across all four correlation models (Supplementary Table 4).

### Correlation analysis of metabolites and cognitive and volumetric measures in HDMCs

At baseline, increased MI was significantly associated with cognitive decline and volumetric reductions. When additionally controlling for CAG repeat length, many of the observed relationships did not reach statistical significance; however, the negative association between MI and caudate volume (*r* = −0.41, *P* < 0.01) remained. Several additional relationships survived across all models, with reduced levels of tNAA being associated with reduced caudate volume (*r* = 0.37, *P* < 0.01) and SCN score (*r* = 0.31, *P* = 0.04), and both tCre and GSH displaying a positive relationship with caudate volume (tCre, *r* = 0.45, *P* < 0.01; GSH, *r* = 0.40, *P* < 0.01) (Fig. 3; Supplementary Table 4).

At follow-up, two relationships observed at baseline were replicated, with tCre (*r* = 0.52, *P* < 0.01) and MI (*r* = −0.44. *P* < 0.01) continuing to display significant correlations with caudate volume across all four models (Fig. 3; Supplementary Table 4).

### Correlation analysis of metabolites and established biofluid biomarkers in HDMCs

At baseline, MI displayed strong positive correlations with CSF and plasma NfL, with the latter remaining significant when additionally controlling for CAG repeat length (*r* = 0.46, *P* < 0.01). GABA also displayed a significant negative association with mHTT (*r* = −0.37, *P* < 0.01) (Fig. 3; Supplementary Table 4).

Cross-sectional analysis at follow-up did not replicate any of the findings observed at baseline. Additional relationships were revealed however, with GLX and tCho displaying significant inverse correlations with mHTT (*r* = −0.54, *P* < 0.01) and CSF tau (*r* = −0.40, *P* < 0.01), respectively. Most notably, negative associations between tCre and multiple biofluid markers were observed across all models (mHTT, *r* = −0.50, *P* < 0.01; CSF NfL, *r* = −0.56, *P* < 0.01; plasma NfL, *r* = −0.49, *P* < 0.01) (Supplementary Table 4).

### Longitudinal analysis of metabolites

Of the 56 subjects at baseline, 42 had longitudinal data available at 24-month follow-up. Nine subjects were removed due to high %SD values in GABA, resulting in a smaller sample size (*n* = 33) for this metabolite.

**Figure 4:**
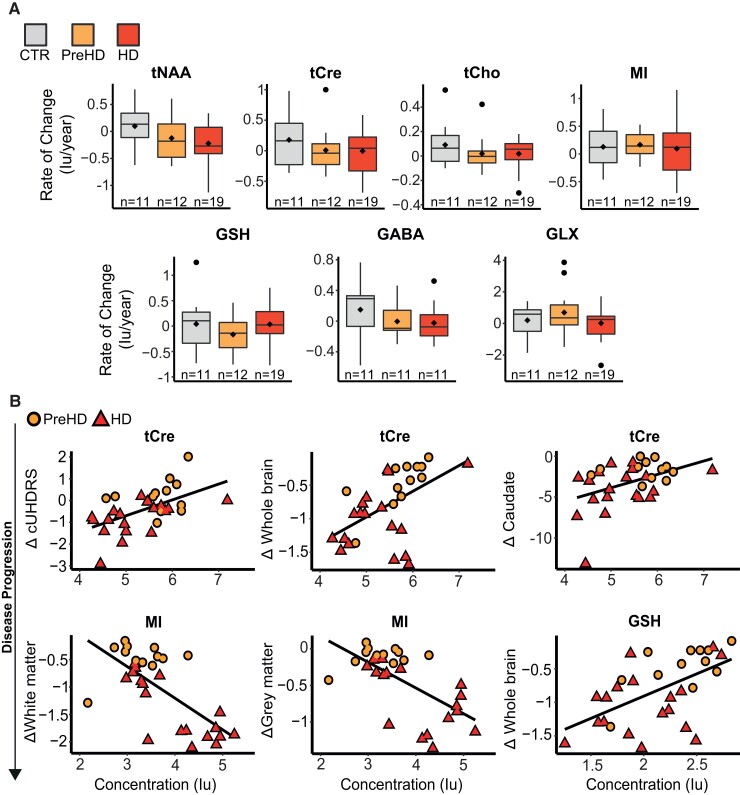
**Longitudinal analysis of ^1^H-MRS metabolites**. (**A**) General linear modes controlling for age, CSF PVE and CAG repeat length revealed no significant differences in annualized rate of change across groups. Tests were not corrected for multiple comparisons. (**B**) tCre correlated with change in cUHDRS (*r* = 0.47, *P* < 0.01), whole brain (*r* = 0.43, *P* = 0.03) and caudate volume (*r* = 0.39, *P* < 0.01), MI significantly predicted decline in white matter (*r* = −0.47, *P* < 0.01) and grey matter volume (*r* = −0.40, *P* < 0.01) and GSH was associated with change in whole brain volume (*r* = 0.39, *P* = 0.03) across all four correlation models. Scatter plots show uncorrected values and contain data from HDMCs only.

The rate of change did not differ across disease stage for any of the primary metabolites. For the secondary metabolites, we found HD to display a greater rate of change in GLX compared with PreHD; however, the regression model did not reach statistical significance (Table 2, Fig. 4A).

To assess prognostic value of the metabolites, we examined if baseline values predicted subsequent change in established measures of disease progression in HDMCs. When controlling for all covariates, we found tCre to display a significant positive correlation with change in cUHDRS (*r* = 0.47, *P* < 0.01), whole brain (*r* = 0.43, *P* = 0.03) and caudate volume (*r* = 0.39, *P* < 0.01), indicating predictive power independent of the core genetic mutation (Fig. 4, Supplementary Table 5). Several additional relationships were also observed, most notably with MRI measures; however, only three remained significant across all 4 correlation models, with MI significantly predicting decline in white matter (*r* = −0.47, *P* < 0.01) and grey matter volume (*r* = −0.40, *P* < 0.01) and GSH associating with change in whole brain volume (*r* = 0.39, *P* = 0.03) (Fig. 4B, Supplementary Table 5).

To assess if rate of change in metabolites provided additional prognostic behaviour beyond that observed using baseline values, we correlated metabolite rate of change, with the rate of change in markers of disease progression in HDMCs. We did not observe any significant relationships that were replicated across all 4 models (Supplementary Table 5).

Mixed-effects models, controlling for age and CSF PVE, revealed GLX to display significant longitudinal change in HDMCs (Table 3), characterized by a slow linear reduction over time.

**Table 3:** Longitudinal trajectory of ^1^H-MRS metabolites

	CTR		HDMCs	
	β	95% CIs	β	95% CIs
**Primary metabolites**				
tNAA	−0.01	−0.20, 0.20	0.07	−0.20, 0.10
tCre	0.10	−0.10, 0.20	−0.10	−0.20, 0.10
tCho	0.10	−0.00, 0.10	0.20	−0.30, 0.10
MI	**0**.**30**	0.10, 0.40	0.10	−0.20, 0.30
**Secondary metabolites**				
GSH	0.10	−0.10, 0.30	−0.10	−0.20, 0.00
GABA	0.00	−0.10, 0.10	0.01	−0.10, 0.00
GLX	−0.20	−0.70, 0.30	**−0**.**40**	−0.80, −0.01

Longitudinal trajectories of all metabolites were studied in CTRs and HDMCs, with GLX displaying a slow linear reduction in HDMCs only [β = −0.40, 95% CIs = (−0.80, −0.01)]. Beta values and 95% confidence intervals were generated from generalized mixed-effects models controlling for age and CSF PVE and have been multiplied by 10 to show change/10yrs. Bold text indicates significance at *P* < 0.05.

## Discussion

In this study, we employed 3 T magnetic resonance spectroscopy to successfully quantify seven metabolites in the putamen of Huntington’s disease patients and CTRs. We specified the most prominent metabolites in the ^1^H spectrum—tNAA, tCre, tCho and MI—as primary metabolites and included lesser studied metabolites—GABA, GLX and GSH—as secondary metabolites. In keeping with previous work, metabolites were normalized to unsuppressed water signal,^25, 28, 37, 62^ allowing for increased accuracy when identifying changes in brain biochemistry^62^ and additionally controlled for CSF PVE. Using general linear models and correlation analysis, we assessed their potential as prognostic and diagnostic biomarkers, both cross-sectionally and longitudinally, by exploring their relationships with established markers of disease progression, cognitive decline, and brain atrophy. Furthermore, we studied the relationship between ^1^H-MRS metabolites and several biomarkers derived from CSF and plasma, including NfL and the pathogenic protein, mHTT. To our knowledge, such relationships have not been explored in Huntington’s disease patients.

When controlling for all covariates, we observed no consistent group differences in metabolite concentration at baseline and follow-up. This finding contrasts with those of Sturrock *et al.*,^25, 37^ who also conducted a ^1^H-MRS analysis of the putamen, in a larger cohort of Huntington’s disease patients, and found multiple metabolites to display significant concentration differences across time points. At baseline, we observed reduced tNAA in HD compared to PreHD; however, the overall model was not significant. At follow-up, the model was significant, and remained so when additionally controlling for CAG repeat length. *Post hoc* tests revealed HD to have significantly lower tNAA concentration compared with PreHD. Similar findings were observed for tCre, which was found to be significantly reduced in HD compared with PreHD at follow-up. Reduced Cr has been consistently demonstrated in Huntington’s disease patients^25, 28, 35^ and may demonstrate diagnostic potential but will require further study in larger samples. Furthermore, Sturrock *et al.* found MI to be increased in HD compared with PreHD at baseline, 12- and 24-month follow-up. Our MI results did not support this and may reflect methodological differences between the studies, specifically the inclusion of age, CSF PVE and CAG repeat length as covariates in all models.

Our study did not find any significant group differences when comparing PreHD to CTRs. This supports earlier work,^25, 26, 34^ in addition to an exploratory study leveraging 7 T MRI,^35^ in which concentrations of Cr, Cho, MI, tNAA, GLX and Lac did not differ in the putamen of PreHD and CTRs, in addition to four other distinct brain regions. Our findings may reflect the fact that our PreHD group were clinically well, demonstrating no significant differences in clinical, cognitive, or volumetric measurements compared with CTRs, whereas other studies may have included PreHD participants closer to clinical onset or with prodromal disease.

In our cross-sectional correlation analysis in HDMCs, MI was significantly associated with caudate volume at both baseline and follow-up. To our knowledge, the relationship observed between MI and plasma NfL at baseline represents the first data in Huntington’s disease patients relating non-invasive ^1^H-MRS measures to an established biofluid marker of disease progression and further highlights the relationship between increased neuroinflammatory response and neurodegenerative processes in Huntington’s disease. MI reflects astrocytic density, while NfL reflects neuro-axonal injury from any mechanism.^63–65^ This association is indicative of astrocytic involvement in neuroinflammation or in compensating for neurodegeneration.^49–68^ However, this finding was not replicated at follow-up and will require further study to better elucidate the relationship between the two measures.

We also observed tCre to be significantly associated with caudate volume at baseline and follow-up, representing the second correlation to be replicated across time points in HDMCs. At follow-up, tCre was also associated with measures of disease progression, neurodegeneration, cognitive decline and biofluid markers, further highlighting the relationship between reduced tCre and a more severe disease phenotype.^28, 35^ Reduced GLX was associated with multiple markers, including CSF mHTT, in the follow-up cohort only. Previous work has shown reduced GLX in the putamen of Huntington’s disease patients and its associations with worse performance on the SDMT.^35^ The lack of multiplicity testing means we cannot rule out false positives in this study and the lack of consistency between both cross-sectional correlational analyses should be acknowledged; however, these results lend support to the notion that creatine concentration may reflect disease activity in a meaningful way, concordant with many other disease measures, and independently of known predictors, and provides additional evidence for reduced GLX being indicative of a worsening clinical phenotype.

Our longitudinal analysis in HDMCs revealed baseline values of tCre to significantly predict subsequent change in cUHDRS, a composite clinical measure sensitive to clinical change,^53^ grey matter and caudate volume. All relationships remained significant when additionally controlling for CAG repeat length. While this predictive potential is of interest, it must be considered in the context of many statistical tests and should therefore be considered exploratory or hypothesis-generating. Furthermore, we found baseline MI values to associate with annualized rate of change in grey and white matter volume. The latter relationship lends support to earlier work highlighting the link between inflammation and myelin breakdown in HD^69^ and demonstrates MI’s potential as a marker of axonal degeneration. Interestingly, we also observed a significant relationship between reduced baseline GSH and larger rate of change in whole brain volume. GSH is a major antioxidant known to be dysregulated in Huntington’s disease^70^ and given that glial cell activation has been linked to increased reactive oxygen species production,^71^ this finding could represent a cyclic cascade of events whereby increased reactive oxygen species production due to neuroinflammation is insufficiently buffered by GSH, resulting in oxidative stress and mitochondrial dysfunction, further driving inflammatory pathways and contributing to the neuropathological hallmarks of the disease.

Mixed-effects models exploring the longitudinal dynamics of all metabolites revealed GLX concentration to reduce linearly with age in HDMCs. No such relationship was observed in CTRs, which may indicate clinical relevance if one was to monitor this biomarker against a reference range derived from a healthy cohort. However, given the lack of group differences, inconsistent correlation results and a protocol not specifically optimized for GLX quantification, this result should be interpreted with caution and further validation is required.

This study is not without its limitations. Due to the exploratory nature of the study, we chose not to adjust our analyses for multiple comparisons. In doing so, we cannot rule out the influence of false positives on our findings, thus our results should be interpreted with caution and further validation is required in future studies. Our decision to adopt more rigorous methodologies also increases the chance of Type 2 (false negative) error but lends greater credibility to our findings overall. Although our results provide some evidence supporting the prognostic potential of specific ^1^H-MRS metabolites, there was a lack of consistency between time points, with only tCre and MI’s association with caudate volume meeting all pre-defined tests at baseline and follow-up. Consequently, further validation is required in a larger sample. Although HD-CSF is a high-quality longitudinal cohort with biofluid collection and MRI imaging, the sample was principally designed to study manifest Huntington’s disease. Previous ^1^H-MRS studies often compared HD participants, or a combination of HD and PreHD, directly to CTRs, explored different brain regions and in some cases, normalized values to metabolites thought to be affected in Huntington’s disease,^28, 30–32^ thus our results may not be directly comparable to earlier work. We acknowledge a limitation of the study related to the use of modelled macromolecular spectra in LCM fitting, rather than experimentally measured spectra as suggested in the recent consensus paper.^72^ Using modelled macromolecular spectra could result in macromolecular components differentiating between studied cohorts, and may also affect the quantitation of GLX, GSH and GABA. Consequently, any results relating to these metabolites should be interpreted with caution. Additionally, as a means of quality control, we excluded some participants based on SNR and %SD values, resulting in a smaller sample size, and reducing the generalisability of the findings. The longitudinal nature of this study is also limited by the small number of available time points. Future studies should aim to incorporate additional time points to help better characterise the longitudinal trajectory of metabolites and improve the models designed to inform on clinical prognosis.

In conclusion, we found no reproducible groupwise differences in metabolite concentration when comparing HD to PreHD and PreHD to CTRs. However, in keeping with previous work, we highlighted the propensity of tNAA and tCre to be reduced in those with advanced disease. This does not exclude the role of ^1^H-MRS-detectable metabolic dysfunctions in disease pathology, only that their use a state biomarker is limited. We found interesting cross-sectional associations between multiple metabolites, namely tCre, MI and GLX, and markers of disease progression, highlighting the proposed roles of neuroinflammation and metabolic dysfunction in Huntington’s disease pathogenesis, but the inconsistent findings between time points and rigorous statistical modelling suggests these changes will have limited biomarker potential. We provide the first evidence, to our knowledge, of an association between ^1^H-MRS metabolites and established CSF biomarkers in HDMCs and found tCre and MI to significantly predict change in measures of disease progression, independent of existing predictors. The potential of non-invasive ^1^H-MRS measurements of brain metabolic activity to monitor the progression of Huntington’s disease or the response to therapeutic interventions warrants directed study of these hypotheses in larger longitudinal imaging cohorts linked to biofluid collection, such as the nascent image-clarity study, which will add advanced imaging modalities to the large, multi-site HDClarity CSF collection initiative.^73^

## Supplementary Material

fcac258_Supplementary_Data

## Abbreviations

Cho =: choline
Cr =: creatine
CTR =: healthy controls
cUHDRS =: composite Unified Huntington’s Disease Rating Scale
DBS =: disease burden score
DCL =: diagnostic confidence level
Glu =: glutamate
GLX =: glutamine + glutamate
GSH =: glutathione
HD =: manifest gene expansion carriers
HDMCs =: Huntington’s disease mutation carriers
^1^H-MRS =: proton magnetic resonance spectroscopy
Iu =: institutional units
Lac =: lactate
LCModel =: linear combination of model spectra
mHTT =: mutant Huntingtin
MI =: myo-inositol
NfL =: neurofilament light chain
ppm =: parts per million
PreHD =: premanifest gene expansion carriers
PVE =: partial volume effect
SD =: standard deviation
SDMT =: Symbol Digit Modality Test
SNR =: signal-to-noise ratio
SWR =: Stroop Word Reading
tCho =: total choline (phosphocholine + glycophosphocholine)
tCre =: total creatine (creatine + phosphocreatine)
TFC =: Total Functional Capacity
TIV =: total intracranial volume
TMS =: total motor score
tNAA =: total *n*-acetylaspartate (*n*-acetylaspartate + *n*-acetylaspartate-glutamate)
VFC =: verbal fluency (categorical)
