# Supplementary material for: Longitudinal evaluation of proton magnetic resonance spectroscopy metabolites as biomarkers in Huntington’s disease
Source: Brain Commun. 2022 Oct 12;4(6):fcac258. doi: 10.1093/braincomms/fcac258 (PMC9665272; doi:10.1093/braincomms/fcac258)
Supplement: fcac258_Supplementary_Data [file fcac258_supplementary_data.docx]

## Supplementary Table 1: MRS metabolite quality control at baseline and follow-up.

| **Metabolites** | **% SD > 100 (n)** | **% SD**  **(Mean)** | **% SD**  **(Max)** | **SD > 20%**  **(% of n)** |
| --- | --- | --- | --- | --- |
| **Primary Metabolites** | | | |  |
| tNAA | 0 | 4.8 | 10 | 0 |
| Follow-up | 0 | 5.1 | 7 | 0 |
| tCre | 0 | 3.4 | 6 | 0 |
| Follow-up | 0 | 3.5 | 7 | 0 |
| tCho | 0 | 4.1 | 7 | 0 |
| Follow-up | 0 | 4.2 | 9 | 0 |
| MI | 0 | 6.5 | 16 | 0 |
| Follow-up | 0 | 6.2 | 13 | 0 |
| **Secondary Metabolites** | | | |  |
| GSH | 0 | 10.9 | 21 | 0.56 |
| Follow-up | 0 | 12.2 | 39 | 1.9 |
| GABA | 8 | 47.7 | 99 | 100 |
| Follow-up | 6 | 45.8 | 99 | 100 |
| GLX | 0 | 7.9 | 17 | 0 |
| Follow-up | 0 | 7.9 | 16 | 0 |

Metabolite measurements with a %SD of >= 100 were removed from all analysis. GABA was the only metabolite with %SD values of >= 100, resulting in the removal of 8 and 6 participants from the baseline and follow-up cohort, respectively. For the remaining metabolites, mean %SD values were <20, indicating high reliability. tNAA, total N‐acetylaspartate; tCr, total creatine; tCho, total choline; MI, myo-inositol; GSH, Glutathione; GLX, glutamine and glutamate.


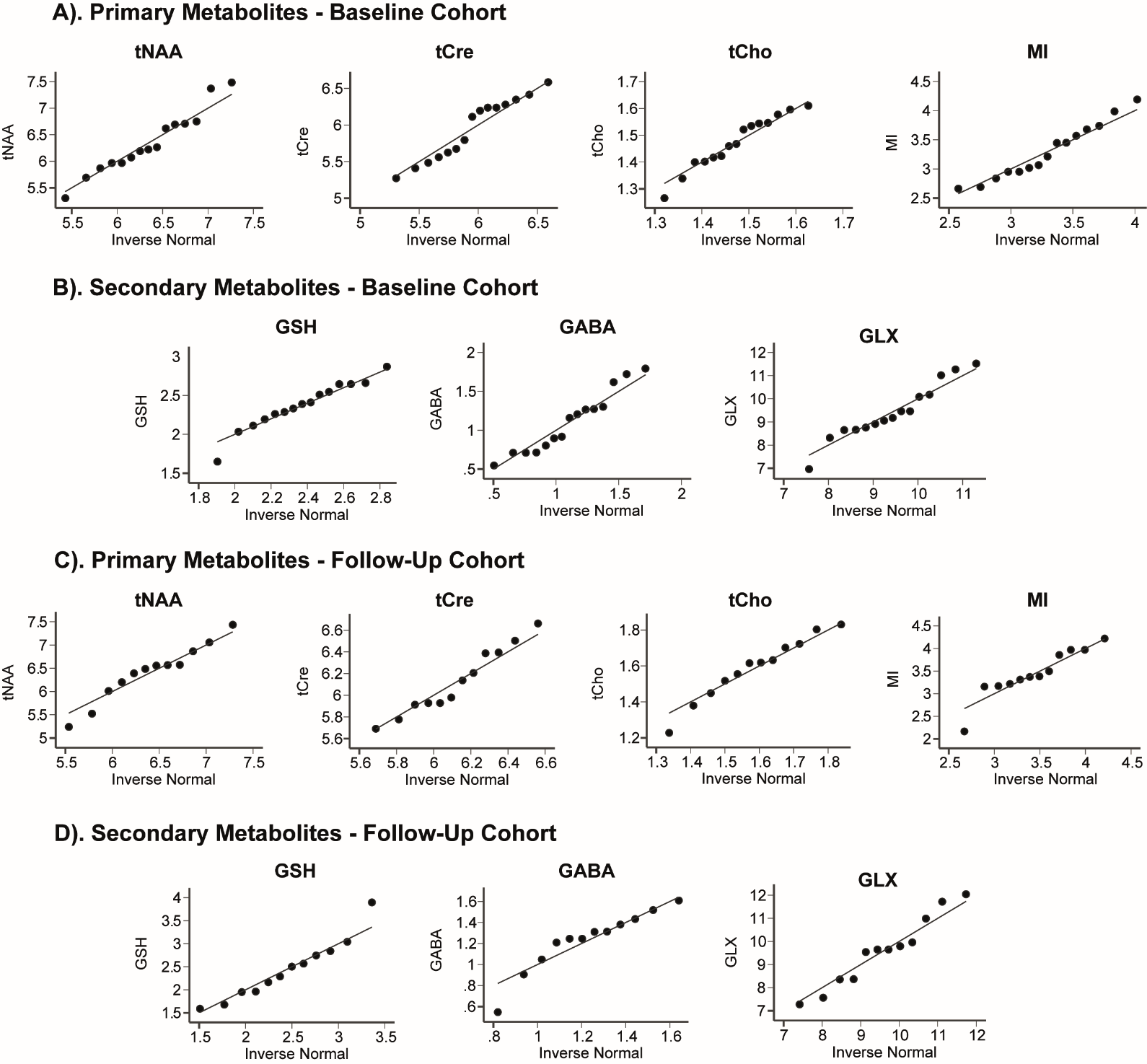


**Supplementary Fig.1: QQ Plots for all metabolites at baseline and follow-up.** QQ plots obtained from controls only. Neither the primary, nor secondary metabolites required data transformation to achieve a suitable distribution.

**Supplementary Table 2. Participant group demographics at baseline and follow-up.**

|  | **CTR** | | **PreHD** | | **HD** | | **Model**  ***p*-value** | **CTR vs PreHD** | **PreHD vs HD** |
| --- | --- | --- | --- | --- | --- | --- | --- | --- | --- |
|  | **n** | **Mean ± SD** | **n** | **Mean ± SD** | **n** | **Mean ± SD** |  |  |  |
| **Demographics** | | | | | | | | | |
| Age (Yrs) | 15 | 49.2 ± 10.5 | 15 | 42.6 ± 11.8 | 26 | 55.7 ± 9.2 | 0.001 | 0.08 | <0.001 |
| Follow-up | 12 | 55.5 ± 10.7 | 13 | 44.7 ± 12.3 | 23 | 58.7 **±** 9.0 | 0.001 | 0.01 | <0.001 |
| Sex (M/F) | 15 | 8/7 | 15 | 8/7 | 26 | 14/12 | 0.99 | N/A | N/A |
| Follow-up | 12 | 8/4 | 13 | 7/6 | 23 | 11/12 | 0.57 | N/A | N/A |
| CAG | N/A | N/A | 15 | 42.1 ± 1.6 | 26 | 42.5 ± 1.8 | 0.48 | N/A | 0.48 |
| Follow-up | N/A | N/A | 13 | 42 ± 1.7 | 23 | 42.5 ± 1.9 | 0.41 | N/A | 0.41 |
| **Clinical Scores (Parentheses indicate observed range)** | | | | | | | | | |
| cUHDRS | 15 | 17.5 ± 1.4 (15.9 - 19.8) | 15 | 18.0 ± 1.2 (16.3 - 20.4) | 26 | 11.1 ± 3.7 (5.6 – 17.9) | <0.001 | 0.62 | <0.001 |
| Follow-up | 12 | 17.4 ± 1.4 (15.6 – 20.4) | 13 | 18.3 ± 1.7 (14.9 – 22.0) | 22 | 9.7 ± 4.7 (2.9 – 17.8) | <0.001 | 0.49 | <0.001 |
| DBS | N/A | N/A | 15 | 275.4 ± 69.2 (116.6 – 388.4) | 26 | 382.5 ± 84.5 (248.6 – 587.3) | <0.001 | N/A | <0.001 |
| Follow-up | N/A | N/A | 13 | 280.4 ± 64.4 (124.5 – 364.0) | 23 | 403.6 ± 98.2 (256.4 – 626.2) | <0.001 | N/A | <0.001 |
| TFC | 15 | 13.0 ± 0.0 (13 – 13) | 15 | 13.0 ± 0.0 (13 – 13) | 26 | 9.8 ± 2.7 (4 – 13) | <0.001 | 1.00 | <0.001 |
| Follow-up | 12 | 13 ± 0 (13 – 13) | 13 | 12.9 ± 0.3 (12 – 13) | 23 | 8.4 ± 3.7 (2 – 13) | <0.001 | 0.94 | <0.001 |
| TMS | 15 | 1.8 ± 1.5 (0 – 5) | 15 | 2.5 ± 2.7 (0 – 8) | 26 | 32.8 ± 16.6 (3 – 56) | <0.001 | 0.86 | <0.001 |
| Follow-up | 12 | 1.9 ± 1.8 (0 – 5) | 13 | 3.3 ± 2.9 (0 – 10) | 23 | 40.5 ± 25.0 (7 – 81) | <0.001 | 0.84 | <0.001 |
| **Cognitive Scores (Parentheses indicate observed range)** | | | | | | | | | |
| SDMT | 15 | 50.9 ± 10.2 (30 – 67) | 15 | 55.5 ± 9.9 (42 – 70) | 26 | 30.0 ± 13.4 (8 – 56) | <0.001 | 0.29 | <0.001 |
| Follow-up | 12 | 51.3 ± 10.1 (34 – 67) | 13 | 58.7 ± 10.7 (46 – 80) | 22 | 26.8 ± 14.9 (6 – 55) | <0.001 | 0.15 | <0.001 |
| SWR | 15 | 101.4 ± 17.9 (73 – 135) | 15 | 105.0 ± 13.1 (84 – 134) | 26 | 64.8 ± 23.5 (18 – 111) | <0.001 | 0.62 | <0.001 |
| Follow-up | 12 | 98.2 ± 16.5 (75 – 135) | 13 | 107.9 ± 21.6 (69 – 146) | 22 | 57.2 ± 26.1 (19 – 122) | <0.001 | 0.29 | <0.001 |
| VFC | 15 | 23.9 ± 4.3 (15 – 30) | 15 | 23.7 ± 3.0 (17 – 27) | 26 | 15.3 ± 6.1 (5 – 26) | <0.001 | 0.91 | <0.001 |
| Follow-up | 12 | 25 ± 4.9 (18 – 36) | 13 | 23.9 ± 5.0 (16 – 36) | 22 | 14.0 ± 6.2 (5 – 25) | <0.001 | 0.63 | <0.001 |
| SCN | 15 | 75.1 ± 13.2 (53 – 104) | 15 | 81.5 ± 11.6 (62 – 105) | 26 | 48.3 ± 17.0 (20 - 90) | <0.001 | 0.24 | <0.001 |
| Follow-up | 12 | 76.2 ± 12.4 (56 – 96) | 13 | 80.8 ± 12.2 (64 – 102) | 22 | 43.9 ± 19.7 (13 – 86) | <0.001 | 0.48 | <0.001 |
| **Volumetric Measures (mL, adjusted for total intracranial volume)** | | | | | | | | | |
| Whole brain | 15 | 1195.0 ± 56.4 | 15 | 1187.5 ± 51.3 | 25 | 1062.2 ± 73.7 | <0.001 | 0.75 | <0.001 |
| Follow-up | 11 | 1191.0 ± 53.1 | 11 | 1176.8 ± 56.3 | 22 | 1048.2 ± 78.4 | <0.001 | 0.62 | <0.001 |
| Caudate volume | 12 | 7.1 ± 0.8 | 14 | 6.1 ± 1.2 | 24 | 3.9 ± 1.1 | <0.001 | 0.02 | <0.001 |
| Follow-up | 11 | 6.9 ± 0.7 | 12 | 6.1 ± 1.0 | 22 | 3.6 ± 1.2 | <0.001 | 0.09 | <0.001 |
| Grey matter | 15 | 705.2 ± 53.0 | 15 | 709.5 ± 48.0 | 26 | 602.8 ± 59.4 | <0.001 | 0.83 | <0.001 |
| Follow-up | 10 | 709.9 ± 43.7 | 12 | 714.1 ± 47.9 | 21 | 592.8 ± 66.2 | <0.001 | 0.86 | <0.001 |
| White matter | 15 | 438.5 ± 34.3 | 15 | 430.4 ± 29.1 | 26 | 386.0 ± 36.3 | <0.001 | 0.52 | <0.001 |
| Follow-up | 10 | 429.4 ± 32.8 | 12 | 419.2 ± 24.3 | 21 | 378.4 ± 40.4 | <0.001 | 0.50 | 0.002 |
| **Biofluid Measures (log pg/ml, unless stated otherwise)** | | | | | | | | | |
| CSF NfL | 15 | 5.9 ± 0.6 | 15 | 6.8 ± 0.8 | 26 | 7.7 ± 0.4 | <0.001 | <0.001 | <0.001 |
| Follow-up | 11 | 6.3 ± 0.7 | 13 | 6.9 ± 0.7 | 22 | 8.0 ± 0.5 | <0.001 | <0.05 | <0.001 |
| CSF mHTT (fM) | N/A | N/A | 15 | 35.0 ± 21.1 | 26 | 50.3 ± 22.8 | <0.05 | N/A | <0.05 |
| Follow-up | N/A | N/A | 12 | 34.6 ± 12.2 | 22 | 60.2 ± 29.8 | <0.01 | N/A | <0.01 |
| CSF Tau | 15 | 4.2 ± 0.3 | 15 | 4.3 ± 0.3 | 26 | 4.5 ± 0.4 | <0.05 | 0.62 | <0.05 |
| Follow-up | 11 | 4.4 ± 0.3 | 13 | 4.4 ± 0.3 | 22 | 4.9 ± 0.4 | <0.001 | 0.86 | <0.01 |
| Plasma NfL | 15 | 1.9 ± 0.4 | 15 | 2.4 ±0.6 | 26 | 3.3 ± 0.4 | <0.001 | <0.01 | <0.001 |
| Follow-up | 12 | 2.2 ± 0.5 | 12 | 2.5 ± 0.6 | 23 | 3.4 ± 0.4 | <0.001 | 0.25 | <0.001 |
| Plasma Tau | 15 | 1.7 ± 0.4 | 15 | 1.6 ± 0.4 | 26 | 1.6 ± 0.5 | 0.81 | 0.71 | 0.81 |
| Follow-up | 12 | 1.9 ± 0.3 | 13 | 2.1 ± 0.2 | 23 | 2.1 ± 0.3 | 0.15 | 0.08 | 0.85 |

Intergroup differences were assessed using general linear models and Pearson’s chi squared test (Gender). P-values are not adjusted for multiple comparisons. Models do not control for age or CAG repeat length. CTR, healthy controls; PreHD, premanifest gene expansion carriers; HD, manifest gene expansion carriers; cUHDRS, composite Unified Huntington’s Disease Rating Scale; DBS, Disease Burden Score; TFC, Total Functional Capacity; TMS, Total Motor Score; SDMT, Symbol Digit Modalities Test; SWR, Stroop Word Reading Test; VFC, Verbal Fluency Categorical; SCN, Stroop Colour Naming; NfL, Neurofilament light; mHTT, mutant Huntingtin; NA, not applicable.

## Supplementary Table 3: Assessments for confounding variables in all metabolites

| **Baseline** | **Age** | | **Gender (CTR)** | | **Gender (HDMC)** | |
| --- | --- | --- | --- | --- | --- | --- |
|  | ***r*** | ***p* value** | ***t*** | ***p* value** | ***t*** | ***p value*** |
| **Primary Metabolites (Iu)** | | | | |  | |
| tNAA | 0.09 | 0.74 | 0.70 | 0.50 | 1.43 | 0.16 |
| Follow-up | 0.10 | 0.75 | 0.19 | 0.85 | 1.31 | 0.20 |
| tCre | -0.01 | 0.97 | **-2.35** | **0.04** | 0.56 | 0.58 |
| Follow-up | 0.29 | 0.36 | -0.57 | 0.58 | 1.37 | 0.18 |
| tCho | 0.18 | 0.51 | -2.16 | 0.04 | -1.35 | 0.19 |
| Follow-up | 0.57 | 0.05 | 0.46 | 0.66 | 0.19 | 0.85 |
| MI | **0.64** | **0.01** | -1.21 | 0.25 | -0.87 | 0.39 |
| Follow-up | 0.53 | 0.08 | 0.15 | 0.88 | -0.30 | 0.76 |
| **Secondary Metabolites (Iu)** | | | | |  |  |
| GSH | 0.52 | 0.05 | -1.02 | 0.33 | 0.36 | 0.72 |
| Follow-up | -0.02 | 0.95 | -0.31 | 0.76 | 0.05 | 0.96 |
| GABA | -0.02 | 0.94 | -0.06 | 0.95 | 0.13 | 0.90 |
| Follow-up | 0.07 | 0.84 | 1.72 | 0.12 | 0.67 | 0.51 |
| GLX | -0.26 | 0.34 | **-2.62** | **0.02** | -0.36 | 0.72 |
| Follow-up | -0.20 | 0.54 | 0.15 | 0.88 | -0.20 | 0.85 |

Values are Pearson’s r and t-test statistic. Bold indicates significance at the P < 0.05 level. Iu, Institutional units; tNAA, total N‐acetylaspartate; tCr, total creatine; tCho, total choline; MI, myo-inositol; GSH, Glutathione; GLX, glutamine and glutamate; CTR, Controls; HDMC, Huntington’s disease mutation carriers.

**Supplementary Table 4 – Correlations between metabolites and clinical, cognitive, volumetric and biofluid measures in baseline and follow-up cohorts.**

|  | | **Age, PVE- adjusted** | | | | | **Age, PVE and**  **CAG-adjusted** | | | | |
| --- | --- | --- | --- | --- | --- | --- | --- | --- | --- | --- | --- |
| **tNAA** | | **Inverse weighted** | | **Bootstrapped** | | | **Inverse weighted** | | **Bootstrapped** | | |
| **Measures** | **n** | **r** | ***p* value** | **r** | **95 % CIs** | ***p* value** | **r** | ***p* value** | **r** | **95 % CIs** | ***p* value** |
| cUHDRS | 41 | 0.25 | 0.12 | 0.27 | -0.05, 0.54 | 0.09 | 0.22 | 0.17 | 0.19 | -0.06, 0.52 | 0.22 |
| Follow-up | 35 | 0.18 | 0.30 | 0.22 | -0.06, 0.46 | 0.10 | 0.36 | **0.04** | 0.39 | 0.13, 0.59 | **<0.01** |
| DBS | 41 | -0.12 | 0.47 | -0.18 | -0.42, 0.08 | 0.17 | NA | NA | NA | NA | NA |
| Follow-up | 36 | 0.08 | 0.65 | 0.04 | -0.23, 0.30 | 0.78 | NA | NA | NA | NA | NA |
| TFC | 41 | 0.21 | 0.20 | 0.22 | -0.11, 0.49 | 0.15 | 0.16 | 0.32 | 0.16 | -0.15, 0.44 | 0.30 |
| Follow-up | 36 | 0.05 | 0.79 | 0.10 | -0.20, 0.37 | 0.49 | 0.13 | 0.44 | 0.19 | -0.09, 0.44 | 0.15 |
| TMS | 41 | -0.24 | 0.13 | -0.24 | -0.53, 0.08 | 0.14 | -0.21 | 0.18 | -0.17 | -0.54, 0.09 | 0.30 |
| Follow-up | 36 | -0.17 | 0.32 | -0.22 | -0.46, 0.05 | 0.10 | -0.31 | 0.07 | -0.35 | -0.59, -0.07 | **<0.01** |
| SDMT | 41 | 0.25 | 0.11 | 0.27 | -0.04, 0.54 | 0.08 | 0.22 | 0.17 | 0.20 | -0.05, 0.51 | 0.17 |
| Follow-up | 35 | 0.23 | 0.18 | 0.27 | -0.04, 0.55 | 0.07 | 0.38 | **0.03** | 0.40 | 0.14, 0.65 | **<0.01** |
| SCN | 41 | 0.37 | **0.02** | 0.38 | 0.11, 0.63 | **<0.01** | 0.35 | **0.02** | 0.31 | -0.05, 0.65 | **0.04** |
| Follow-up | 35 | 0.19 | 0.26 | 0.22 | -0.08, 0.46 | 0.11 | 0.37 | **0.03** | 0.39 | 0.11, 0.60 | **<0.01** |
| VFC | 41 | 0.34 | **0.03** | 0.33 | 0.04, 0.58 | **0.02** | 0.35 | **0.03** | 0.27 | 0.02, 0.59 | 0.07 |
| Follow-up | 35 | 0.07 | 0.68 | 0.08 | -0.18, 0.34 | 0.52 | 0.16 | 0.35 | 0.17 | -0.15, 0.46 | 0.26 |
| SWR | 41 | 0.23 | 0.15 | 0.24 | -0.05, 0.51 | 0.10 | 0.18 | 0.27 | 0.14 | -0.07, 0.46 | 0.35 |
| Follow-up | 35 | 0.22 | 0.20 | 0.24 | -0.12, 0.46 | 0.05 | 0.38 | **0.02** | 0.39 | 0.15, 0.59 | **<0.01** |
| Whole brain | 40 | 0.30 | 0.06 | 0.24 | -0.03, 0.48 | 0.07 | 0.24 | 0.14 | 0.18 | -0.06, 0.42 | 0.15 |
| Follow-up | 34 | 0.16 | 0.38 | 0.16 | -0.17, 0.40 | 0.25 | 0.19 | 0.29 | 0.20 | -0.09, 0.47 | 0.15 |
| Caudate | 38 | 0.39 | **0.02** | 0.37 | 0.10, 0.64 | **<0.01** | 0.42 | **<0.01** | 0.37 | 0.12, 0.67 | **<0.01** |
| Follow-up | 34 | 0.24 | 0.18 | 0.23 | -0.05, 0.46 | 0.08 | 0.34 | 0.05 | 0.33 | 0.08, 0.55 | **<0.01** |
| White matter | 41 | 0.12 | 0.44 | 0.03 | -0.24, 0.31 | 0.82 | 0.14 | 0.38 | 0.04 | -0.23, 0.29 | 0.74 |
| Follow-up | 33 | -0.02 | 0.93 | 0.00 | -0.26, 0.26 | 0.98 | -0.01 | 0.97 | 0.02 | -0.24, 0.28 | 0.91 |
| Grey matter | 41 | 0.43 | **<0.01** | 0.36 | 0.02, 0.57 | **0.01** | 0.44 | **<0.01** | 0.33 | -0.04, 0.61 | 0.06 |
| Follow-up | 33 | 0.22 | 0.21 | 0.24 | -0.09, 0.53 | 0.13 | 0.26 | 0.14 | 0.29 | -0.03, 0.60 | 0.08 |
| CSF NfL (log) | 41 | -0.18 | 0.27 | -0.22 | -0.45, 0.02 | 0.08 | -0.08 | 0.60 | -0.07 | -0.32, 0.16 | 0.56 |
| Follow-up | 35 | -0.06 | 0.75 | -0.07 | -0.35, 0.23 | 0.65 | -0.16 | 0.34 | -0.18 | -0.45, 0.17 | 0.26 |
| CSF Tau (log) | 41 | -0.03 | 0.86 | -0.08 | -0.42, 0.25 | 0.65 | 0.02 | 0.91 | -0.03 | -0.34, 0.26 | 0.84 |
| Follow-up | 35 | 0.16 | 0.35 | 0.14 | -0.12, 0.39 | 0.26 | 0.14 | 0.44 | 0.12 | -0.13, 0.39 | 0.34 |
| CSF mHTT | 41 | -0.14 | 0.40 | -0.14 | -0.37, 0.13 | 0.30 | -0.05 | 0.78 | 0.03 | -0.34, 0.31 | 0.88 |
| Follow-up | 34 | -0.00 | 0.99 | -0.04 | -0.32, 0.26 | 0.81 | -0.08 | 0.64 | -0.10 | -0.40, 0.20 | 0.53 |
| Plasma NfL (log) | 41 | -0.28 | 0.08 | -0.29 | -0.59, 0.04 | 0.07 | -0.25 | 0.11 | -0.22 | -0.59, 0.01 | 0.15 |
| Follow-up | 36 | -0.11 | 0.51 | -0.11 | -0.40, 0.17 | 0.42 | -0.25 | 0.14 | -0.26 | -0.51, 0.11 | 0.10 |
| Plasma Tau (log) | 41 | -0.29 | 0.06 | -0.29 | -0.53, 0.07 | **0.02** | -0.29 | 0.07 | -0.31 | -0.51, -0.08 | **<0.01** |
| Follow-up | 36 | 0.07 | 0.69 | 0.05 | -0.29, 0.38 | 0.78 | 0.04 | 0.83 | 0.02 | -0.35, 0.34 | 0.91 |

|  | | **Age, PVE- adjusted** | | | | | **Age, PVE and**  **CAG-adjusted** | | | | |
| --- | --- | --- | --- | --- | --- | --- | --- | --- | --- | --- | --- |
| **tCre** | | **Inverse weighted** | | **Bootstrapped** | | | **Inverse weighted** | | **Bootstrapped** | | |
| **Measures** | **n** | **r** | ***p* value** | **r** | **95 % CIs** | ***p* value** | **r** | ***p* value** | **r** | **95 % CIs** | ***p* value** |
| cUHDRS | 41 | 0.17 | 0.28 | 0.17 | -0.16, 0.45 | 0.26 | 0.27 | 0.09 | 0.26 | -0.05, 0.53 | 0.08 |
| Follow-up | 35 | 0.53 | **<0.01** | 0.49 | 0.13, 0.71 | **<0.01** | 0.43 | **<0.01** | 0.40 | 0.12, 0.64 | **<0.01** |
| DBS | 41 | 0.10 | 0.54 | 0.07 | -0.16, 0.29 | 0.51 | NA | NA | NA | NA | NA |
| Follow-up | 36 | -0.38 | **0.02** | -0.33 | -0.63, 0.02 | 0.04 | NA | NA | NA | NA | NA |
| TFC | 41 | 0.02 | 0.88 | 0.07 | -0.20, 0.35 | 0.64 | 0.05 | 0.73 | 0.11 | -0.19, 0.43 | 0.50 |
| Follow-up | 36 | 0.34 | **0.04** | 0.35 | 0.03, 0.65 | **0.03** | 0.14 | 0.43 | 0.19 | -0.10, 0.51 | 0.23 |
| TMS | 41 | -0.13 | 0.42 | -0.11 | -0.40, 0.20 | 0.48 | -0.20 | 0.21 | -0.17 | -0.46, 0.14 | 0.25 |
| Follow-up | 36 | -0.57 | **<0.01** | -0.52 | -0.75, 0.20 | **<0.01** | -0.49 | **<0.01** | -0.44 | -0.69, -0.16 | **<0.01** |
| SDMT | 41 | 0.26 | 0.10 | 0.26 | -0.09, 0.51 | 0.09 | 0.35 | **0.02** | 0.34 | 0.03, 0.59 | **0.02** |
| Follow-up | 35 | 0.51 | **<0.01** | 0.45 | 0.14, 0.65 | **<0.01** | 0.41 | **0.01** | 0.36 | 0.10, 0.58 | **<0.01** |
| SCN | 41 | 0.30 | 0.05 | 0.29 | -0.06, 0.55 | 0.06 | 0.41 | **<0.01** | 0.40 | 0.07, 0.63 | **<0.01** |
| Follow-up | 35 | 0.56 | **<0.01** | 0.51 | 0.18, 0.71 | **<0.01** | 0.48 | **<0.01** | 0.44 | 0.18, 0.67 | **<0.01** |
| VFC | 41 | 0.17 | 0.29 | 0.14 | -0.14, 0.43 | 0.33 | 0.28 | 0.08 | 0.24 | -0.04, 0.49 | 0.07 |
| Follow-up | 35 | 0.41 | **0.01** | 0.36 | 0.06, 0.57 | **<0.01** | 0.30 | 0.08 | 0.27 | -0.06, 0.53 | 0.07 |
| SWR | 41 | 0.18 | 0.25 | 0.17 | -0.16, 0.44 | 0.26 | 0.28 | 0.08 | 0.26 | -0.06, 0.51 | 0.07 |
| Follow-up | 35 | 0.52 | **<0.01** | 0.47 | 0.09, 0.69 | **<0.01** | 0.42 | **0.01** | 0.38 | 0.02, 0.62 | **<0.01** |
| Whole brain | 40 | 0.25 | 0.12 | 0.18 | -0.12, 0.44 | 0.21 | 0.28 | 0.08 | 0.21 | -0.05, 0.45 | 0.11 |
| Follow-up | 34 | 0.39 | **0.02** | 0.36 | 0.05, 0.73 | 0.05 | 0.40 | **0.02** | 0.36 | -0.02, 0.70 | 0.05 |
| Caudate | 38 | 0.43 | **<0.01** | 0.37 | 0.07, 0.62 | **<0.01** | 0.53 | **<0.01** | 0.45 | 0.14, 0.65 | **<0.01** |
| Follow-up | 34 | 0.50 | **<0.01** | 0.47 | 0.20, 0.75 | **<0.01** | 0.57 | **<0.01** | 0.52 | 0.30, 0.76 | **<0.01** |
| White matter | 41 | 0.17 | 0.28 | 0.13 | -0.15, 0.36 | 0.33 | 0.18 | 0.27 | 0.13 | -0.13, 0.36 | 0.32 |
| Follow-up | 33 | 0.26 | 0.14 | 0.21 | -0.22, 0.57 | 0.29 | 0.25 | 0.16 | 0.20 | -0.26, 0.55 | 0.34 |
| Grey matter | 41 | 0.30 | 0.06 | 0.20 | -0.12, 0.45 | 0.18 | 0.37 | **0.02** | 0.25 | -0.10, 0.50 | 0.09 |
| Follow-up | 33 | 0.44 | **0.01** | 0.42 | 0.15, 0.67 | **<0.01** | 0.45 | **<0.01** | 0.44 | 0.11, 0.68 | **<0.01** |
| CSF NfL (log) | 41 | -0.18 | 0.25 | -0.12 | -0.43, 0.18 | 0.43 | -0.29 | 0.06 | -0.21 | -0.47, 0.19 | 0.20 |
| Follow-up | 35 | -0.64 | **<0.01** | -0.57 | -0.76, -0.25 | **<0.01** | -0.62 | **<0.01** | -0.56 | -0.75, -0.26 | **<0.01** |
| CSF Tau (log) | 41 | -0.06 | 0.72 | -0.01 | -0.35, 0.52 | 0.97 | -0.07 | 0.68 | -0.02 | -0.37, 0.52 | 0.94 |
| Follow-up | 35 | -0.45 | **<0.01** | -0.38 | -0.67, -0.09 | **0.01** | -0.29 | 0.09 | -0.25 | -0.53, 0.00 | 0.07 |
| CSF mHTT | 41 | -0.09 | 0.58 | -0.00 | -0.33, 0.39 | 0.98 | -0.15 | 0.35 | -0.05 | -0.40, 0.46 | 0.83 |
| Follow-up | 34 | -0.57 | **<0.01** | -0.54 | -0.78, -0.24 | **<0.01** | -0.49 | **<0.01** | -0.50 | -0.78, -0.22 | **<0.01** |
| Plasma NfL (log) | 41 | -0.27 | 0.09 | -0.23 | -0.50, 0.06 | 0.12 | -0.40 | **<0.01** | -0.35 | -0.58, -0.11 | **<0.01** |
| Follow-up | 36 | -0.61 | **<0.01** | -0.55 | -0.76, 0.21 | **<0.01** | -0.54 | **<0.01** | -0.49 | -0.75, -0.23 | **<0.01** |
| Plasma Tau (log) | 41 | -0.23 | 0.15 | -0.28 | -0.52, 0.03 | **0.04** | -0.23 | 0.15 | -0.28 | -0.52, 0.01 | 0.05 |
| Follow-up | 36 | -0.06 | 0.71 | -0.07 | -0.37, 0.18 | 0.65 | 0.11 | 0.53 | 0.07 | -0.19, 0.29 | 0.57 |

|  | | **Age, PVE-**  **adjusted** | | | | | **Age, PVE and**  **CAG-adjusted** | | | | |
| --- | --- | --- | --- | --- | --- | --- | --- | --- | --- | --- | --- |
| **tCho** | | **Inverse weighted** | | **Bootstrapped** | | | **Inverse weighted** | | **Bootstrapped** | | |
| **Measures** | **n** | **r** | ***p* value** | **r** | **95 % CIs** | ***p* value** | **r** | ***p* value** | **r** | **95 % CIs** | ***p* value** |
| cUHDRS | 41 | -0.10 | 0.55 | -0.08 | -0.39, 0.24 | 0.62 | 0.10 | 0.54 | 0.10 | -0.19, 0.35 | 0.46 |
| Follow-up | 35 | 0.20 | 0.26 | 0.11 | -0.28, 0.44 | 0.56 | 0.15 | 0.40 | 0.11 | -0.24, 0.41 | 0.48 |
| DBS | 41 | 0.33 | **0.04** | 0.33 | 0.08, 0.51 | **<0.01** | NA | NA | NA | NA | NA |
| Follow-up | 36 | -0.12 | 0.50 | -0.00 | -0.42, 0.33 | 0.99 | NA | NA | NA | NA | NA |
| TFC | 41 | -0.20 | 0.22 | -0.15 | -0.43, 0.21 | 0.33 | -0.07 | 0.63 | -0.03 | -0.32, 0.29 | 0.82 |
| Follow-up | 36 | -0.07 | 0.70 | -0.12 | -0.45, 0.23 | 0.50 | -0.21 | 0.21 | -0.19 | -0.45, 0.09 | 0.19 |
| TMS | 41 | 0.14 | 0.39 | 0.12 | -0.21, 0.42 | 0.46 | -0.02 | 0.89 | -0.03 | -0.31, 0.29 | 0.85 |
| Follow-up | 36 | -0.24 | 0.16 | -0.14 | -0.44, 0.22 | 0.42 | -0.21 | 0.22 | -0.16 | -0.41, 0.21 | 0.32 |
| SDMT | 41 | 0.03 | 0.88 | 0.04 | -0.30, 0.37 | 0.83 | 0.22 | 0.16 | 0.22 | -0.07, 0.46 | 0.10 |
| Follow-up | 35 | 0.27 | 0.11 | 0.18 | -0.20, 0.48 | 0.30 | 0.25 | 0.15 | 0.20 | -0.13, 0.51 | 0.21 |
| SCN | 41 | -0.04 | 0.79 | -0.05 | -0.32, 0.24 | 0.72 | 0.17 | 0.28 | 0.16 | -0.07, 0.38 | 0.17 |
| Follow-up | 35 | 0.26 | 0.13 | 0.17 | -0.18, 0.48 | 0.34 | 0.24 | 0.17 | 0.20 | -0.10, 0.53 | 0.2 |
| VFC | 41 | 0.08 | 0.60 | 0.08 | -0.21, 0.37 | 0.60 | 0.41 | **<0.01** | 0.38 | 0.14, 0.55 | **<0.01** |
| Follow-up | 35 | 0.15 | 0.38 | 0.09 | -0.20, 0.40 | 0.54 | 0.09 | 0.62 | 0.09 | -0.20, 0.42 | 0.55 |
| SWR | 41 | -0.09 | 0.56 | -0.10 | -0.36, 0.21 | 0.50 | 0.12 | 0.46 | 0.10 | -0.16, 0.34 | 0.44 |
| Follow-up | 35 | 0.25 | 0.15 | 0.17 | -0.18, 0.47 | 0.33 | 0.21 | 0.22 | 0.19 | -0.09, 0.48 | 0.19 |
| Whole brain | 40 | 0.09 | 0.57 | 0.03 | -0.29, 0.39 | 0.85 | 0.20 | 0.22 | 0.13 | -0.16, 0.52 | 0.43 |
| Follow-up | 34 | 0.07 | 0.68 | 0.04 | -0.34, 0.36 | 0.82 | 0.14 | 0.41 | 0.11 | -0.27, 0.42 | 0.57 |
| Caudate | 38 | 0.01 | 0.93 | -0.05 | -0.36, 0.30 | 0.79 | 0.13 | 0.45 | 0.04 | -0.30, 0.40 | 0.82 |
| Follow-up | 34 | 0.04 | 0.84 | -0.00 | -0.31, 0.40 | 0.99 | 0.18 | 0.31 | 0.13 | -0.20, 0.53 | 0.48 |
| White matter | 41 | 0.26 | 0.11 | 0.23 | -0.10, 0.49 | 0.13 | 0.28 | 0.08 | 0.24 | -0.05, 0.78 | 0.08 |
| Follow-up | 33 | 0.14 | 0.43 | 0.11 | -0.23, 0.46 | 0.53 | 0.19 | 0.28 | 0.16 | -0.21, 0.50 | 0.40 |
| Grey matter | 41 | 0.11 | 0.49 | 0.00 | -0.40, 0.34 | 0.99 | 0.26 | 0.09 | 0.13 | -0.30, 0.48 | 0.52 |
| Follow-up | 33 | 0.07 | 0.72 | 0.04 | -0.32, 0.35 | 0.83 | 0.13 | 0.47 | 0.10 | -0.29, 0.39 | 0.57 |
| CSF NfL (log) | 41 | 0.28 | 0.07 | 0.32 | -0.02, 0.55 | **0.01** | 0.13 | 0.42 | 0.18 | -0.07, 0.42 | 0.16 |
| Follow-up | 35 | -0.42 | **0.01** | -0.33 | -0.60, 0.06 | 0.05 | -0.48 | **<0.01** | -0.43 | -0.66, -0.12 | **<0.01** |
| CSF Tau (log) | 41 | -0.04 | 0.79 | -0.02 | -0.32, 0.34 | 0.89 | -0.11 | 0.48 | -0.08 | -0.35, 0.34 | 0.61 |
| Follow-up | 35 | -0.41 | **0.02** | -0.40 | -0.65, -0.13 | **<0.01** | -0.38 | **0.03** | -0.40 | -0.61, -0.11 | **<0.01** |
| CSF mHTT | 41 | 0.03 | 0.86 | 0.09 | -0.23, 0.40 | 0.58 | -0.20 | 0.19 | -0.13 | -0.43, 0.25 | 0.46 |
| Follow-up | 34 | -0.38 | **0.03** | -0.33 | -0.64, 0.02 | 0.06 | -0.40 | **0.02** | -0.39 | -0.62, -0.08 | **<0.01** |
| Plasma NfL (log) | 41 | 0.18 | 0.25 | 0.19 | -0.15, 0.45 | 0.22 | 0.01 | 0.93 | 0.04 | -0.27, 0.31 | 0.81 |
| Follow-up | 36 | -0.36 | **0.03** | -0.23 | -0.58, 0.14 | 0.23 | -0.40 | **0.02** | -0.31 | -0.60, 0.01 | 0.06 |
| Plasma Tau (log) | 41 | -0.20 | 0.20 | -0.25 | -0.49, 0.02 | 0.05 | -0.21 | 0.17 | -0.27 | -0.48, -0.00 | **0.02** |
| Follow-up | 36 | 0.05 | 0.77 | 0.08 | -0.24, 0.36 | 0.61 | 0.10 | 0.58 | 0.10 | -0.19, 0.36 | 0.48 |

|  | | **Age, PVE-**  **adjusted** | | | | | **Age, PVE and**  **CAG-adjusted** | | | | |
| --- | --- | --- | --- | --- | --- | --- | --- | --- | --- | --- | --- |
| **MI** | | **Inverse weighted** | | **Bootstrapped** | | | **Inverse weighted** | | **Bootstrapped** | | |
| **Measures** | **n** | **r** | ***p* value** | **r** | **95 % CIs** | ***p* value** | **r** | ***p* value** | **r** | **95 % CIs** | ***p* value** |
| cUHDRS | 41 | -0.33 | **0.04** | -0.38 | -0.60, -0.10 | **<0.01** | -0.20 | 0.21 | -0.28 | -0.57, 0.01 | 0.05 |
| Follow-up | 35 | -0.19 | 0.28 | -0.24 | -0.49, 0.06 | 0.08 | -0.22 | 0.21 | -0.23 | -0.49, -0.01 | 0.07 |
| DBS | 41 | 0.22 | 0.16 | 0.33 | -0.02, 0.55 | 0.02 | NA | NA | NA | NA | NA |
| Follow-up | 36 | -0.02 | 0.90 | 0.08 | -0.20, 0.28 | 0.53 | NA | NA | NA | NA | NA |
| TFC | 41 | -0.20 | 0.21 | -0.24 | -0.50, 0.01 | 0.07 | -0.05 | 0.75 | -0.12 | -0.41, 0.18 | 0.41 |
| Follow-up | 36 | -0.16 | 0.34 | -0.23 | -0.51, 0.17 | 0.18 | -0.16 | 0.35 | -0.21 | -0.47, 0.18 | 0.20 |
| TMS | 41 | 0.32 | **0.04** | 0.42 | 0.08, 0.64 | **<0.01** | 0.20 | 0.20 | 0.36 | -0.04, 0.63 | **0.04** |
| Follow-up | 36 | 0.17 | 0.31 | 0.20 | -0.05, 0.44 | 0.11 | 0.18 | 0.28 | 0.17 | -0.10, 0.44 | 0.22 |
| SDMT | 41 | -0.30 | 0.06 | -0.34 | -0.58, -0.00 | **0.02** | -0.18 | 0.27 | -0.23 | -0.48, 0.06 | 0.09 |
| Follow-up | 35 | -0.10 | 0.56 | -0.16 | -0.43, 0.12 | 0.24 | -0.09 | 0.60 | -0.12 | -0.39, 0.10 | 0.33 |
| SCN | 41 | -0.34 | **0.03** | -0.40 | -0.63, -0.16 | **<0.01** | -0.20 | 0.21 | -0.27 | -0.62, 0.01 | 0.09 |
| Follow-up | 35 | -0.24 | 0.17 | -0.28 | -0.56, -0.04 | **0.03** | -0.29 | 0.09 | -0.29 | -0.49, 0.07 | **<0.01** |
| VFC | 41 | -0.31 | 0.05 | -0.37 | -0.61, -0.07 | **<0.01** | -0.15 | 0.36 | -0.24 | -0.52, 0.07 | 0.12 |
| Follow-up | 35 | -0.15 | 0.39 | -0.22 | -0.44, 0.09 | 0.10 | -0.17 | 0.34 | -0.20 | -0.42, 0.02 | 0.09 |
| SWR | 41 | -0.38 | **0.01** | -0.40 | -0.62, -0.18 | **<0.01** | -0.25 | 0.11 | -0.28 | -0.59, -0.04 | **0.04** |
| Follow-up | 35 | -0.25 | 0.15 | -0.28 | -0.50, -0.04 | **0.02** | -0.29 | 0.09 | -0.28 | -0.49, -0.07 | **0.01** |
| Whole brain | 40 | -0.24 | 0.14 | -0.35 | -0.55, 0.07 | **<0.01** | -0.19 | 0.25 | -0.31 | -0.53, -0.00 | **0.02** |
| Follow-up | 34 | -0.29 | 0.09 | -0.35 | -0.57, -0.02 | **<0.01** | -0.32 | 0.06 | -0.34 | -0.53, -0.05 | **<0.01** |
| Caudate | 38 | -0.40 | **0.01** | -0.43 | -0.64, -0.17 | **<0.01** | -0.38 | **0.02** | -0.41 | -0.68, -0.14 | **<0.01** |
| Follow-up | 34 | -0.38 | **0.03** | -0.42 | -0.69, -0.17 | **<0.01** | -0.45 | **<0.01** | -0.44 | -0.63, -0.21 | **<0.01** |
| White matter | 41 | -0.04 | 0.79 | -0.14 | -0.39, 0.19 | 0.34 | -0.07 | 0.65 | -0.17 | -0.41, 0.40 | 0.19 |
| Follow-up | 33 | 0.02 | 0.92 | -0.09 | -0.36, 0.23 | 0.57 | 0.04 | 0.83 | -0.06 | -0.37, 0.22 | 0.69 |
| Grey matter | 41 | -0.31 | 0.05 | -0.44 | -0.63, 0.19 | **<0.01** | -0.25 | 0.11 | -0.39 | -0.61, -0.11 | **<0.01** |
| Follow-up | 33 | -0.46 | **<0.01** | -0.50 | -0.70, -0.20 | **<0.01** | -0.51 | **<0.01** | -0.52 | -0.69, -0.23 | **<0.01** |
| CSF NfL (log) | 41 | 0.33 | **0.04** | 0.34 | 0.10, 0.56 | **<0.01** | 0.15 | 0.36 | 0.14 | -0.14, 0.43 | 0.33 |
| Follow-up | 35 | 0.22 | 0.20 | 0.23 | -0.04, 0.54 | 0.13 | 0.16 | 0.34 | 0.14 | -0.10, 0.40 | 0.26 |
| CSF Tau (log) | 41 | 0.08 | 0.63 | 0.05 | -0.35, 0.44 | 0.80 | -0.01 | 0.96 | -0.02 | -0.38, 0.34 | 0.93 |
| Follow-up | 35 | 0.18 | 0.29 | 0.15 | -0.15, 0.42 | 0.30 | 0.10 | 0.59 | 0.06 | -0.24, 0.31 | 0.68 |
| CSF mHTT | 41 | 0.11 | 0.48 | 0.20 | -0.25, 0.46 | 0.25 | -0.14 | 0.38 | -0.03 | -0.43, 0.27 | 0.85 |
| Follow-up | 34 | 0.14 | 0.44 | 0.17 | -0.11, 0.46 | 0.25 | -0.00 | 0.99 | 0.03 | -0.25, 0.26 | 0.82 |
| Plasma NfL (log) | 41 | 0.49 | **<0.01** | 0.50 | 0.18, 0.71 | **<0.01** | 0.45 | **<0.01** | 0.46 | 0.10, 0.72 | **<0.01** |
| Follow-up | 36 | 0.15 | 0.37 | 0.20 | -0.08, 0.53 | 0.21 | 0.17 | 0.32 | 0.18 | -0.15, 0.43 | 0.22 |
| Plasma Tau (log) | 41 | 0.04 | 0.80 | -0.06 | -0.41, 0.33 | 0.75 | 0.04 | 0.79 | -0.06 | -0.41, 0.33 | 0.75 |
| Follow-up | 36 | 0.19 | 0.27 | 0.17 | -0.23, 0.36 | 0.18 | 0.16 | 0.36 | 0.13 | -0.24, 0.36 | 0.35 |

|  | | **Age, PVE-**  **adjusted** | | | | | **Age, PVE and**  **CAG-adjusted** | | | | |
| --- | --- | --- | --- | --- | --- | --- | --- | --- | --- | --- | --- |
| **GSH** | | **Inverse weighted** | | **Bootstrapped** | | | **Inverse weighted** | | **Bootstrapped** | | |
| **Measures** | **n** | **r** | ***p* value** | **r** | **95 % CIs** | ***p* value** | **r** | ***p* value** | **r** | **95 % CIs** | ***p* value** |
| cUHDRS | 41 | 0.09 | 0.56 | 0.17 | -0.12, 0.42 | 0.24 | 0.15 | 0.35 | 0.21 | -0.08, 0.48 | 0.14 |
| Follow-up | 35 | 0.24 | 0.16 | 0.26 | -0.11, 0.54 | 0.12 | 0.01 | 0.94 | 0.06 | -0.23, 0.35 | 0.71 |
| DBS | 41 | 0.02 | 0.89 | 0.00 | -0.32, 0.33 | 0.98 | NA | NA | NA | NA | NA |
| Follow-up | 36 | -0.41 | **0.01** | -0.36 | -0.62, -0.10 | **0.01** | NA | NA | NA | NA | NA |
| TFC | 41 | 0.10 | 0.54 | 0.17 | -0.08, 0.44 | 0.18 | 0.14 | 0.38 | 0.21 | -0.07, 0.49 | 0.14 |
| Follow-up | 36 | 0.22 | 0.20 | 0.21 | -0.13, 0.50 | 0.21 | 0.01 | 0.95 | -0.00 | -0.28, 0.32 | 0.99 |
| TMS | 41 | -0.01 | 0.94 | -0.06 | -0.32, 0.24 | 0.67 | -0.03 | 0.83 | -0.08 | -0.37, 0.22 | 0.62 |
| Follow-up | 36 | -0.28 | 0.10 | -0.31 | -0.60, -0.01 | 0.05 | -0.11 | 0.54 | -0.16 | -0.41, 0.12 | 0.26 |
| SDMT | 41 | 0.12 | 0.46 | 0.19 | -0.11, 0.49 | 0.20 | 0.17 | 0.30 | 0.23 | -0.10, 0.49 | 0.13 |
| Follow-up | 35 | 0.27 | 0.12 | 0.28 | -0.06, 0.55 | 0.08 | 0.10 | 0.57 | 0.12 | -0.18, 0.43 | 0.46 |
| SCN | 41 | 0.18 | 0.27 | 0.22 | -0.02, 0.46 | 0.07 | 0.24 | 0.13 | 0.27 | 0.00, 0.48 | **0.02** |
| Follow-up | 35 | 0.14 | 0.43 | 0.18 | -0.18, 0.50 | 0.33 | -0.14 | 0.43 | -0.07 | -0.37, 0.29 | 0.70 |
| VFC | 41 | 0.10 | 0.54 | 0.13 | -0.17, 0.41 | 0.40 | 0.16 | 0.31 | 0.17 | -0.13, 0.43 | 0.24 |
| Follow-up | 35 | 0.22 | 0.20 | 0.23 | -0.17, 0.58 | 0.23 | 0.06 | 0.75 | 0.08 | -0.33, 0.43 | 0.69 |
| SWR | 41 | 0.11 | 0.49 | 0.18 | -0.07, 0.43 | 0.18 | 0.17 | 0.29 | 0.22 | -0.03, 0.48 | 0.09 |
| Follow-up | 35 | 0.09 | 0.61 | 0.14 | -0.24, 0.46 | 0.44 | -0.18 | 0.30 | -0.10 | -0.38, 0.21 | 0.50 |
| Whole brain | 40 | 0.23 | 0.15 | 0.24 | -0.07, 0.51 | 0.11 | 0.25 | 0.11 | 0.26 | -0.02, 0.53 | 0.07 |
| Follow-up | 34 | 0.08 | 0.65 | 0.24 | -0.03, 0.54 | 0.12 | 0.06 | 0.70 | 0.22 | -0.08, 0.50 | 0.15 |
| Caudate | 38 | 0.35 | **0.03** | 0.36 | 0.07, 0.59 | **<0.01** | 0.41 | **0.01** | 0.40 | 0.11, 0.61 | **<0.01** |
| Follow-up | 34 | 0.02 | 0.91 | 0.16 | -0.17, 0.45 | 0.32 | -0.08 | 0.65 | 0.08 | -0.25, 0.35 | 0.62 |
| White matter | 41 | 0.16 | 0.31 | 0.19 | -0.07, 0.44 | 0.16 | 0.18 | 0.27 | 0.19 | -0.06, 0.46 | 0.15 |
| Follow-up | 33 | 0.29 | 0.11 | 0.26 | -0.12, 0.55 | 0.14 | 0.27 | 0.13 | 0.23 | -0.09, 0.51 | 0.14 |
| Grey matter | 41 | 0.25 | 0.12 | 0.20 | -0.14, 0.47 | 0.20 | 0.30 | 0.06 | 0.23 | -0.12, 0.48 | 0.13 |
| Follow-up | 33 | -0.01 | 0.94 | 0.22 | -0.15, 0.56 | 0.21 | -0.03 | 0.88 | 0.19 | -0.19, 0.52 | 0.30 |
| CSF NfL (log) | 41 | -0.08 | 0.62 | -0.05 | -0.37. 0.23 | 0.74 | -0.13 | 0.43 | -0.07 | -0.42, 0.21 | 0.68 |
| Follow-up | 35 | -0.28 | 0.11 | -0.30 | -0.60, 0.05 | 0.08 | -0.20 | 0.24 | -0.20 | -0.46, 0.12 | 0.16 |
| CSF Tau (log) | 41 | -0.05 | 0.74 | -0.02 | -0.38, 0.32 | 0.92 | -0.06 | 0.73 | -0.02 | -0.37, 0.32 | 0.92 |
| Follow-up | 35 | -0.19 | 0.27 | -0.19 | -0.50, 0.16 | 0.25 | -0.06 | 0.71 | -0.08 | -0.35, 0.25 | 0.60 |
| CSF mHTT | 41 | 0.05 | 0.76 | 0.09 | -0.22, 0.39 | 0.55 | 0.05 | 0.77 | 0.13 | -0.19, 0.46 | 0.42 |
| Follow-up | 34 | -0.32 | 0.07 | -0.37 | -0.67, 0.01 | **0.04** | -0.25 | 0.16 | -0.29 | -0.57, 0.23 | 0.06 |
| Plasma NfL (log) | 41 | -0.16 | 0.32 | -0.15 | -0.44, 0.13 | 0.31 | -0.24 | 0.13 | -0.20 | -0.49, 0.07 | 0.15 |
| Follow-up | 36 | -0.26 | 0.13 | -0.29 | -0.54, -0.01 | **0.04** | -0.05 | 0.75 | -0.09 | -0.36, 0.19 | 0.55 |
| Plasma Tau (log) | 41 | -0.22 | 0.16 | -0.31 | -0.54, 0.07 | **0.04** | -0.23 | 0.16 | -0.31 | -0.54, 0.06 | **0.04** |
| Follow-up | 36 | -0.15 | 0.41 | -0.17 | -0.45, 0.12 | 0.22 | -0.00 | 0.98 | -0.05 | -0.30, 0.21 | 0.71 |

|  | | **Age, PVE-**  **adjusted** | | | | | **Age, PVE and**  **CAG-adjusted** | | | | |
| --- | --- | --- | --- | --- | --- | --- | --- | --- | --- | --- | --- |
| **GABA** | | **Inverse weighted** | | **Bootstrapped** | | | **Inverse weighted** | | **Bootstrapped** | | |
| **Measures** | **n** | **r** | ***p* value** | **r** | **95 % CIs** | ***p* value** | **r** | ***p* value** | **r** | **95 % CIs** | ***p* value** |
| cUHDRS | 33 | 0.21 | 0.25 | 0.23 | -0.13, 0.51 | 0.17 | 0.19 | 0.29 | 0.20 | -0.06, 0.49 | 0.17 |
| Follow-up | 29 | 0.18 | 0.36 | 0.16 | -0.18, 0.39 | 0.23 | 0.07 | 0.70 | 0.03 | -0.51, 0.35 | 0.87 |
| DBS | 33 | -0.04 | 0.81 | -0.08 | -0.37, 0.28 | 0.63 | NA | NA | NA | NA | NA |
| Follow-up | 30 | -0.18 | 0.34 | -0.19 | -0.64, 0.24 | 0.40 | NA | NA | NA | NA | NA |
| TFC | 33 | 0.09 | 0.60 | 0.15 | -0.13, 0.39 | 0.27 | 0.06 | 0.74 | 0.11 | -0.14, 0.36 | 0.37 |
| Follow-up | 30 | 0.15 | 0.43 | 0.16 | -0.31, 0.40 | 0.34 | 0.01 | 0.98 | 0.01 | -0.40, 0.36 | 0.95 |
| TMS | 33 | -0.17 | 0.34 | -0.18 | -0.50, 0.17 | 0.29 | -0.16 | 0.39 | -0.15 | -0.47, 0.16 | 0.35 |
| Follow-up | 30 | -0.26 | 0.17 | -0.23 | -0.45, 0.06 | 0.07 | -0.15 | 0.43 | -0.12 | -0.40, 0.36 | 0.53 |
| SDMT | 33 | 0.21 | 0.24 | 0.20 | -0.17, 0.49 | 0.22 | 0.19 | 0.29 | 0.17 | -0.12, 0.43 | 0.23 |
| Follow-up | 29 | 0.18 | 0.35 | 0.12 | -0.19, 0.38 | 0.42 | 0.09 | 0.64 | -0.00 | -0.55, 0.34 | 0.99 |
| SCN | 33 | 0.33 | 0.06 | 0.34 | -0.03, 0.58 | **0.03** | 0.32 | 0.07 | 0.30 | -0.00, 0.56 | **0.03** |
| Follow-up | 29 | 0.26 | 0.17 | 0.23 | -0.10, 0.44 | 0.08 | 0.20 | 0.30 | 0.13 | -0.39, 0.43 | 0.52 |
| VFC | 33 | 0.26 | 0.15 | 0.27 | -0.07, 0.55 | 0.09 | 0.25 | 0.16 | 0.24 | -0.07, 0.52 | 0.11 |
| Follow-up | 29 | 0.25 | 0.18 | 0.20 | -0.17, 0.43 | 0.18 | 0.22 | 0.26 | 0.12 | -0.47, 0.46 | 0.59 |
| SWR | 33 | 0.26 | 0.14 | 0.29 | -0.14, 0.58 | 0.09 | 0.25 | 0.16 | 0.27 | -0.05, 0.56 | 0.10 |
| Follow-up | 29 | 0.15 | 0.44 | 0.15 | -0.23, 0.39 | 0.32 | 0.04 | 0.82 | 0.03 | -0.42, 0.37 | 0.88 |
| Whole brain | 32 | 0.11 | 0.56 | 0.12 | -0.15, 0.39 | 0.38 | 0.05 | 0.80 | 0.08 | -0.22, 0.35 | 0.60 |
| Follow-up | 28 | 0.12 | 0.53 | 0.06 | -0.28, 0.35 | 0.71 | 0.04 | 0.84 | -0.00 | -0.35, 0.32 | 0.99 |
| Caudate | 32 | 0.15 | 0.41 | 0.16 | -0.13, 0.47 | 0.29 | 0.14 | 0.45 | 0.14 | -0.26, 0.45 | 0.45 |
| Follow-up | 28 | 0.35 | 0.07 | 0.32 | -0.03, 0.53 | **0.02** | 0.24 | 0.21 | 0.23 | -0.14, 0.53 | 0.15 |
| White matter | 33 | 0.10 | 0.59 | 0.07 | -0.28, 0.38 | 0.69 | 0.13 | 0.48 | 0.08 | -0.24, 0.37 | 0.58 |
| Follow-up | 27 | 0.06 | 0.77 | -0.01 | -0.39, 0.28 | 0.94 | -0.02 | 0.92 | -0.06 | -0.46, 0.23 | 0.71 |
| Grey matter | 33 | 0.35 | 0.05 | 0.31 | -0.06, 0.56 | **0.02** | 0.35 | **0.04** | 0.29 | -0.09, 0.64 | 0.12 |
| Follow-up | 27 | 0.17 | 0.39 | 0.12 | -0.22, 0.37 | 0.40 | 0.09 | 0.65 | 0.07 | -0.24, 0.36 | 0.67 |
| CSF NfL (log) | 33 | -0.20 | 0.27 | -0.19 | -0.47, 0.19 | 0.26 | -0.14 | 0.43 | -0.09 | -0.42, 0.22 | 0.58 |
| Follow-up | 29 | -0.20 | 0.29 | -0.28 | -0.58, 0.05 | 0.09 | -0.08 | 0.69 | -0.19 | -0.43, 0.17 | 0.18 |
| CSF Tau (log) | 33 | -0.26 | 0.14 | -0.28 | -0.56, 0.00 | 0.06 | -0.25 | 0.16 | -0.26 | -0.54, 0.02 | 0.08 |
| Follow-up | 29 | -0.07 | 0.71 | -0.09 | -0.28, 0.24 | 0.48 | 0.05 | 0.78 | 0.02 | -0.23, 0.29 | 0.88 |
| CSF mHTT | 33 | -0.38 | **0.03** | -0.38 | -0.61, -0.10 | **<0.01** | -0.39 | **0.03** | -0.37 | -0.60, -0.05 | **<0.01** |
| Follow-up | 28 | -0.18 | 0.37 | -0.16 | -0.40, 0.16 | 0.24 | -0.11 | 0.59 | -0.16 | -0.42, 0.12 | 0.25 |
| Plasma NfL (log) | 33 | -0.10 | 0.59 | -0.16 | -0.45, 0.15 | 0.29 | -0.06 | 0.74 | -0.12 | -0.42, 0.25 | 0.49 |
| Follow-up | 30 | -0.17 | 0.36 | -0.22 | -0.48, 0.11 | 0.12 | 0.00 | 0.98 | -0.07 | -0.35, 0.22 | 0.62 |
| Plasma Tau (log) | 33 | -0.02 | 0.90 | -0.01 | -0.30, 0.32 | 0.97 | -0.05 | 0.78 | -0.05 | -0.35, 0.27 | 0.77 |
| Follow-up | 30 | 0.13 | 0.50 | 0.16 | -0.31, 0.49 | 0.45 | 0.20 | 0.28 | 0.23 | -0.28, 0.57 | 0.28 |

|  | | **Age, PVE-**  **adjusted** | | | | | **Age, PVE and**  **CAG-adjusted** | | | | |
| --- | --- | --- | --- | --- | --- | --- | --- | --- | --- | --- | --- |
| **GLX** | | **Inverse weighted** | | **Bootstrapped** | | | **Inverse weighted** | | **Bootstrapped** | | |
| **Measures** | **n** | **r** | ***p* value** | **r** | **95 % CIs** | ***p* value** | **r** | ***p* value** | **r** | **95 % CIs** | ***p* value** |
| cUHDRS | 41 | 0.13 | 0.43 | 0.17 | -0.12, 0.44 | 0.29 | 0.14 | 0.40 | 0.14 | -0.14, 0.43 | 0.36 |
| Follow-up | 35 | 0.37 | **0.03** | 0.40 | 0.05, 0.61 | **<0.01** | 0.37 | **0.03** | 0.40 | 0.13, 0.57 | **<0.01** |
| DBS | 41 | -0.10 | 0.53 | -0.12 | -0.38, 0.12 | 0.36 | NA | NA | NA | NA | NA |
| Follow-up | 36 | -0.28 | 0.10 | -0.25 | -0.50, 0.15 | 0.13 | NA | NA | NA | NA | NA |
| TFC | 41 | 0.05 | 0.76 | 0.07 | -0.23, 0.36 | 0.62 | 0.03 | 0.83 | 0.03 | -0.23, 0.37 | 0.85 |
| Follow-up | 36 | 0.30 | 0.08 | 0.32 | -0.06, 0.59 | 0.05 | 0.22 | 0.20 | 0.26 | -0.02, 0.49 | **0.04** |
| TMS | 41 | -0.08 | 0.60 | -0.12 | -0.43, 0.28 | 0.51 | -0.08 | 0.61 | -0.08 | -0.41, 0.27 | 0.65 |
| Follow-up | 36 | -0.46 | **<0.01** | -0.47 | -0.66, -0.21 | **<0.01** | -0.46 | **<0.01** | -0.47 | -0.65, -0.29 | **<0.01** |
| SDMT | 41 | 0.18 | 0.25 | 0.22 | -0.13, 0.48 | 0.16 | 0.20 | 0.21 | 0.20 | -0.11, 0.46 | 0.18 |
| Follow-up | 35 | 0.32 | 0.06 | 0.34 | 0.05, 0.54 | **<0.01** | 0.29 | 0.09 | 0.30 | 0.09, 0.48 | **<0.01** |
| SCN | 41 | 0.16 | 0.33 | 0.18 | -0.08, 0.46 | 0.18 | 0.16 | 0.33 | 0.14 | -0.14, 0.44 | 0.35 |
| Follow-up | 35 | 0.28 | 0.11 | 0.33 | 0.00, 0.57 | **0.02** | 0.23 | 0.18 | 0.30 | -0.05, 0.50 | **0.02** |
| VFC | 41 | 0.24 | 0.13 | 0.28 | -0.04, 0.55 | 0.06 | 0.30 | 0.06 | 0.30 | 0.05, 0.55 | **0.02** |
| Follow-up | 35 | 0.44 | **<0.01** | 0.42 | 0.09, 0.68 | **<0.01** | 0.45 | **<0.01** | 0.43 | 0.09, 0.65 | **<0.01** |
| SWR | 41 | 0.13 | 0.41 | 0.18 | -0.09, 0.45 | 0.20 | 0.13 | 0.40 | 0.15 | -0.06, 0.43 | 0.24 |
| Follow-up | 35 | 0.31 | 0.07 | 0.35 | 0.03, 0.60 | **0.02** | 0.28 | 0.10 | 0.33 | -0.05, 0.56 | **0.02** |
| Whole brain | 40 | 0.11 | 0.49 | 0.14 | -0.22, 0.42 | 0.39 | 0.12 | 0.47 | 0.12 | -0.24, 0.41 | 0.47 |
| Follow-up | 34 | 0.39 | **0.02** | 0.37 | 0.05, 0.61 | **<0.01** | 0.40 | **0.02** | 0.38 | 0.07, 0.61 | **<0.01** |
| Caudate | 38 | 0.24 | 0.15 | 0.29 | -0.08, 0.56 | 0.06 | 0.25 | 0.13 | 0.27 | -0.05, 0.55 | 0.08 |
| Follow-up | 34 | 0.31 | 0.07 | 0.33 | 0.03, 0.61 | **0.03** | 0.31 | 0.07 | 0.34 | 0.06, 0.57 | **0.01** |
| White matter | 41 | 0.15 | 0.33 | 0.18 | -0.12, 0.43 | 0.22 | 0.17 | 0.29 | 0.19 | -0.13, 0.45 | 0.22 |
| Follow-up | 33 | 0.48 | **<0.01** | 0.46 | 0.10, 0.68 | **<0.01** | 0.48 | **<0.01** | 0.46 | 0.15, 0.67 | **<0.01** |
| Grey matter | 41 | 0.01 | 0.97 | 0.02 | -0.43, 0.32 | 0.89 | -0.02 | 0.92 | -0.02 | -0.42, 0.28 | 0.90 |
| Follow-up | 33 | 0.37 | **0.04** | 0.38 | 0.02, 0.63 | **0.02** | 0.37 | **0.03** | 0.39 | 0.01, 0.65 | **0.02** |
| CSF NfL (log) | 41 | -0.02 | 0.88 | -0.04 | -0.35, 0.25 | 0.75 | 0.02 | 0.93 | 0.05 | -0.22, 0.30 | 0.72 |
| Follow-up | 35 | -0.16 | 0.37 | -0.15 | -0.46, 0.17 | 0.36 | -0.09 | 0.62 | -0.10 | -0.38, 0.19 | 0.50 |
| CSF Tau (log) | 41 | -0.15 | 0.35 | -0.19 | -0.60, 0.23 | 0.36 | -0.15 | 0.36 | -0.18 | -0.57, 0.24 | 0.39 |
| Follow-up | 35 | -0.21 | 0.22 | -0.25 | -0.56, 0.09 | 0.14 | -0.16 | 0.36 | -0.21 | -0.49, 0.12 | 0.18 |
| CSF mHTT | 41 | -0.20 | 0.19 | -0.23 | -0.55, 0.13 | 0.18 | -0.23 | 0.14 | -0.21 | -0.48, 0.15 | 0.19 |
| Follow-up | 34 | -0.46 | **<0.01** | -0.47 | -0.67, -0.14 | **<0.01** | -0.53 | **<0.01** | -0.54 | -0.70, -0.26 | **<0.01** |
| Plasma NfL (log) | 41 | -0.10 | 0.54 | -0.15 | -0.46, 0.21 | 0.38 | -0.11 | 0.51 | -0.11 | -0.40, 0.16 | 0.42 |
| Follow-up | 36 | -0.15 | 0.39 | -0.13 | -0.40, 0.15 | 0.35 | 0.00 | 0.98 | 0.02 | -0.18, 0.22 | 0.88 |
| Plasma Tau (log) | 41 | -0.11 | 0.50 | -0.13 | -0.40, 0.12 | 0.31 | -0.12 | 0.45 | -0.14 | -0.40, 0.11 | 0.31 |
| Follow-up | 36 | -0.01 | 0.94 | 0.02 | -0.32, 0.34 | 0.93 | 0.08 | 0.64 | 0.10 | -0.23, 0.41 | 0.55 |

Relationships between MRS metabolites and clinical, cognitive, volumetric and biofluid markers were assessed at baseline and follow-up using Pearson’s partial correlation controlling for age and CSF PVE, and age, CSF PVE and CAG repeat length. When analysing DBS, only CSF PVE was adjusted for. Correlation coefficients and 95% confidence intervals were computed using bootstrap testing with 1000 repetitions. A weighted correlation was also conducted, applying an inverse weighting to %SD value. Results displayed are unadjusted for multiplicity. Bold text indicated significance P < 0.05. cUHDRS, composite Unified Huntington’s Disease Rating Scale; DBS, Disease Burden Score; TFC, Total Functional Capacity; TMS, Total Motor Score; SDMT, Symbol Digit Modalities Test; SCN, Stroop Colour Naming; VFC, Verbal Fluency Categorical; SWR, Stroop Word Reading Test; NfL, Neurofilament Light Chain; mHTT, Mutant Huntingtin: PVE, partial volume effect.

## Supplementary Table 5 – Longitudinal correlations between metabolites and annualised rate of change (Δ) in clinical, cognitive, and volumetric measures.

| **tNAA** | |  | **Age, PVE-adjusted** | | | | | **Age, PVE and**  **CAG-adjusted** | | | | |
| --- | --- | --- | --- | --- | --- | --- | --- | --- | --- | --- | --- | --- |
|  | |  | **Inverse weighted** | | **Bootstrapped** | | | **Inverse weighted** | | **Bootstrapped** | | |
| **Measures** | **n** | **Association with** | **r** | ***p* value** | **r** | **95 % CIs** | ***p* value** | **r** | ***p* value** | **r** | **95 % CIs** | ***p* value** |
| Δ cUHDRS | 31 | Baseline tNAA | 0.28 | 0.12 | 0.27 | -0.06, 0.57 | 0.09 | 0.28 | 0.12 | 0.25 | -0.06, 0.53 | 0.11 |
|  |  | Δ tNAA | 0.08 | 0.68 | 0.05 | -0.31, 0.34 | 0.74 | 0.09 | 0.64 | 0.09 | -0.24, 0.37 | 0.56 |
| Δ DBS | 31 | Baseline tNAA | -0.03 | 0.86 | -0.10 | -0.41, 0.18 | 0.51 | NA | NA | NA | NA | NA |
|  |  | Δ tNAA | 0.11 | 0.53 | 0.15 | -0.21, 0.51 | 0.41 | NA | NA | NA | NA | NA |
| Δ TFC | 31 | Baseline tNAA | -0.02 | 0.91 | 0.01 | -0.36, 0.35 | 0.97 | -0.05 | 0.78 | -0.04 | -0.41, 0.28 | 0.81 |
|  |  | Δ tNAA | 0.00 | 0.99 | 0.02 | -0.29, 0.26 | 0.91 | 0.03 | 0.84 | 0.07 | -0.30, 0.34 | 0.67 |
| Δ TMS | 31 | Baseline tNAA | -0.12 | 0.51 | -0.09 | -0.42, 0.24 | 0.59 | -0.13 | 0.48 | -0.07 | -0.40, 0.26 | 0.65 |
|  |  | Δ tNAA | 0.00 | 0.99 | -0.04 | -0.28, 0.25 | 0.78 | 0.00 | 0.98 | -0.05 | -0.31, 0.24 | 0.70 |
| Δ SDMT | 31 | Baseline tNAA | 0.31 | 0.09 | 0.31 | -0.14, 0.65 | 0.14 | 0.29 | 0.11 | 0.29 | -0.08, 0.63 | 0.13 |
|  |  | Δ tNAA | 0.20 | 0.29 | 0.13 | -0.32, 0.44 | 0.52 | 0.19 | 0.31 | 0.14 | -0.28, 0.41 | 0.45 |
| Δ SCN | 31 | Baseline tNAA | 0.03 | 0.86 | 0.07 | -0.22, 0.45 | 0.68 | 0.02 | 0.92 | 0.04 | -0.26, 0.38 | 0.81 |
|  |  | Δ tNAA | -0.01 | 0.97 | -0.05 | -0.38, 0.36 | 0.78 | -0.02 | 0.91 | -0.05 | -0.35, 0.34 | 0.79 |
| Δ VFC | 31 | Baseline tNAA | 0.27 | 0.14 | 0.21 | -0.14, 0.49 | 0.18 | 0.29 | 0.11 | 0.25 | -0.11, 0.49 | 0.10 |
|  |  | Δ tNAA | -0.02 | 0.92 | -0.04 | -0.45, 0.36 | 0.86 | -0.05 | 0.77 | -0.06 | -0.46, 0.34 | 0.76 |
| Δ SWR | 31 | Baseline tNAA | 0.34 | 0.07 | 0.28 | 0.05, 0.52 | **0.01** | 0.32 | 0.07 | 0.26 | -0.00, 0.47 | **0.03** |
|  |  | Δ tNAA | -0.00 | 0.99 | -0.04 | -0.40, 0.29 | 0.82 | -0.01 | 0.94 | -0.04 | -0.36, 0.26 | 0.83 |
| Δ Whole brain | 30 | Baseline tNAA | 0.46 | **0.01** | 0.44 | 0.18, 0.66 | **<0.01** | 0.34 | 0.07 | 0.35 | 0.08, 0.57 | **<0.01** |
|  |  | Δ tNAA | 0.22 | 0.25 | 0.17 | -0.15, 0.45 | 0.26 | 0.29 | 0.11 | 0.27 | -0.04, 0.55 | 0.08 |
| Δ Caudate | 31 | Baseline tNAA | 0.13 | 0.48 | 0.17 | -0.08, 0.44 | 0.22 | 0.11 | 0.55 | 0.11 | -0.14, 0.38 | 0.39 |
|  |  | Δ tNAA | 0.10 | 0.57 | 0.11 | -0.21, 0.34 | 0.43 | 0.14 | 0.44 | 0.17 | -0.09, 0.37 | 0.13 |
| Δ White matter | 30 | Baseline tNAA | 0.25 | 0.18 | 0.27 | -0.05, 0.59 | 0.11 | 0.34 | 0.21 | 0.21 | -0.06, 0.46 | 0.13 |
|  |  | Δ tNAA | 0.16 | 0.39 | 0.16 | -0.15, 0.50 | 0.32 | 0.24 | 0.21 | 0.27 | -0.05, 0.57 | 0.09 |
| Δ Grey matter | 30 | Baseline tNAA | 0.10 | 0.61 | 0.11 | -0.23, 0.48 | 0.55 | 0.06 | 0.74 | 0.05 | -0.21, 0.37 | 0.74 |
|  |  | Δ tNAA | 0.18 | 0.34 | 0.19 | -0.15, 0.48 | 0.22 | 0.24 | 0.21 | 0.27 | -0.09, 0.53 | 0.08 |

| **tCre** | |  | **Age, PVE-adjusted** | | | | | **Age, PVE and**  **CAG-adjusted** | | | | |
| --- | --- | --- | --- | --- | --- | --- | --- | --- | --- | --- | --- | --- |
|  | |  | **Inverse weighted** | | **Bootstrapped** | | | **Inverse weighted** | | **Bootstrapped** | | |
| **Measures** | **n** | **Association with** | **r** | ***p* value** | **r** | **95 % CIs** | ***p* value** | **r** | ***p* value** | **r** | **95 % CIs** | ***p* value** |
| Δ cUHDRS | 31 | Baseline tCre | 0.46 | **<0.01** | 0.43 | 0.15, 0.61 | **<0.01** | 0.49 | **<0.01** | 0.47 | 0.24, 0.63 | **<0.01** |
|  |  | Δ tCre | 0.03 | 0.87 | -0.03 | -0.31, 0.39 | 0.86 | 0.03 | 0.86 | -0.03 | -0.34, 0.33 | 0.85 |
| Δ DBS | 31 | Baseline tCre | 0.07 | 0.72 | 0.09 | -0.25, 0.38 | 0.59 | NA | NA | NA | NA | NA |
|  |  | Δ tCre | -0.20 | 0.28 | -0.19 | -0.55, 0.23 | 0.33 | NA | NA | NA | NA | NA |
| Δ TFC | 31 | Baseline tCre | 0.36 | 0.05 | 0.35 | -0.03, 0.63 | **0.04** | 0.39 | **0.03** | 0.38 | -0.02, 0.61 | **0.01** |
|  |  | Δ tCre | -0.03 | 0.86 | -0.05 | -0.45, 0.30 | 0.80 | -0.04 | 0.84 | -0.05 | -0.40, 0.30 | 0.77 |
| Δ TMS | 31 | Baseline tCre | -0.30 | 0.10 | -0.30 | -0.52, 0.11 | 0.06 | -0.31 | 0.09 | -0.30 | -0.53, 0.18 | 0.08 |
|  |  | Δ tCre | -0.22 | 0.24 | -0.17 | -0.48, 0.13 | 0.29 | -0.22 | 0.23 | -0.18 | -0.49, 0.17 | 0.31 |
| Δ SDMT | 31 | Baseline tCre | 0.23 | 0.22 | 0.23 | -0.08, 0.50 | 0.15 | 0.22 | 0.22 | 0.23 | -0.08, 0.48 | 0.14 |
|  |  | Δ tCre | -0.00 | 0.99 | -0.07 | -0.41, 0.30 | 0.71 | -0.00 | 0.99 | -0.07 | -0.42, 0.27 | 0.70 |
| Δ SCN | 31 | Baseline tCre | 0.04 | 0.82 | 0.05 | -0.29, 0.40 | 0.79 | 0.04 | 0.84 | 0.05 | -0.32, 0.42 | 0.80 |
|  |  | Δ tCre | 0.09 | 0.65 | 0.03 | -0.37, 0.46 | 0.90 | 0.09 | 0.64 | 0.03 | -0.41, 0.45 | 0.90 |
| Δ VFC | 31 | Baseline tCre | 0.07 | 0.72 | 0.05 | -0.33, 0.44 | 0.80 | 0.07 | 0.71 | 0.05 | -0.31, 0.45 | 0.79 |
|  |  | Δ tCre | 0.12 | 0.50 | 0.06 | -0.44, 0.43 | 0.79 | 0.12 | 0.51 | 0.06 | -0.44, 0.42 | 0.79 |
| Δ SWR | 31 | Baseline tCre | 0.30 | 0.10 | 0.26 | -0.00, 0.49 | **0.03** | 0.30 | 0.10 | 0.26 | 0.01, 0.48 | **0.03** |
|  |  | Δ tCre | -0.07 | 0.72 | -0.09 | -0.31, 0.18 | 0.47 | -0.07 | 0.72 | -0.09 | -0.32, 0.16 | 0.45 |
| Δ Whole brain | 30 | Baseline tCre | 0.43 | **0.02** | 0.40 | -0.04, 0.71 | **0.04** | 0.45 | **0.01** | 0.43 | 0.04, 0.45 | **0.03** |
|  |  | Δ tCre | 0.03 | 0.85 | -0.06 | -0.55, 0.34 | 0.78 | 0.03 | 0.88 | -0.08 | -0.56, 0.35 | 0.73 |
| Δ Caudate | 31 | Baseline tCre | 0.40 | **0.03** | 0.35 | 0.03, 0.58 | **0.02** | 0.44 | **0.01** | 0.39 | 0.03, 0.58 | **<0.01** |
|  |  | Δ tCre | 0.12 | 0.54 | 0.08 | -0.35, 0.42 | 0.69 | 0.13 | 0.48 | 0.09 | -0.28, 0.41 | 0.60 |
| Δ White matter | 30 | Baseline tCre | 0.31 | 0.09 | 0.26 | -0.18, 0.63 | 0.22 | 0.39 | **0.03** | 0.32 | -0.10, 0.61 | 0.08 |
|  |  | Δ tCre | 0.21 | 0.27 | 0.16 | -0.39, 0.50 | 0.47 | 0.24 | 0.21 | 0.19 | -0.27, 0.55 | 0.38 |
| Δ Grey matter | 30 | Baseline tCre | 0.15 | 0.42 | 0.11 | -0.31, 0.43 | 0.57 | 0.17 | 0.36 | 0.12 | -0.20, 0.46 | 0.49 |
|  |  | Δ tCre | 0.31 | 0.09 | 0.28 | -0.08, 0.53 | 0.07 | 0.35 | 0.06 | 0.31 | -0.00, 0.56 | **0.02** |

| **tCho** | |  | **Age, PVE-adjusted** | | | | | **Age, PVE and**  **CAG-adjusted** | | | | |
| --- | --- | --- | --- | --- | --- | --- | --- | --- | --- | --- | --- | --- |
|  | |  | **Inverse weighted** | | **Bootstrapped** | | | **Inverse weighted** | | **Bootstrapped** | | |
| **Measures** | **n** | **Association with** | **r** | ***p* value** | **r** | **95 % CIs** | ***p* value** | **r** | ***p* value** | **r** | **95 % CIs** | ***p* value** |
| Δ cUHDRS | 31 | Baseline tCho | 0.33 | 0.07 | 0.27 | -0.11, 0.55 | 0.12 | 0.45 | **0.01** | 0.40 | 0.03, 0.65 | **0.01** |
|  |  | Δ tCho | 0.01 | 0.98 | -0.03 | -0.29, 0.33 | 0.83 | -0.04 | 0.85 | -0.08 | -0.38, 0.24 | 0.59 |
| Δ DBS | 31 | Baseline tCho | 0.28 | 0.11 | 0.30 | -0.02, 0.58 | 0.05 | NA | NA | NA | NA | NA |
|  |  | Δ tCho | -0.32 | 0.08 | -0.32 | -0.54, 0.00 | **0.03** | NA | NA | NA | NA | NA |
| Δ TFC | 31 | Baseline tCho | 0.21 | 0.26 | 0.16 | -0.30, 0.51 | 0.47 | 0.32 | 0.08 | 0.25 | -0.16, 0.59 | 0.20 |
|  |  | Δ tCho | 0.04 | 0.86 | 0.04 | -0.22, 0.29 | 0.74 | -0.04 | 0.82 | -0.03 | -0.26, 0.19 | 0.76 |
| Δ TMS | 31 | Baseline tCho | -0.31 | 0.09 | -0.22 | -0.58, 0.21 | 0.31 | -0.35 | 0.06 | -0.26 | -0.59, 0.18 | 0.21 |
|  |  | Δ tCho | 0.05 | 0.80 | 0.04 | -0.22, 0.28 | 0.78 | 0.07 | 0.72 | 0.06 | -0.18, 0.35 | 0.65 |
| Δ SDMT | 31 | Baseline tCho | 0.10 | 0.58 | 0.10 | -0.24, 0.37 | 0.53 | 0.15 | 0.42 | 0.15 | -0.20, 0.53 | 0.42 |
|  |  | Δ tCho | 0.07 | 0.69 | -0.00 | -0.32, 0.38 | 0.99 | 0.07 | 0.71 | -0.01 | -0.37, 0.33 | 0.96 |
| Δ SCN | 31 | Baseline tCho | -0.08 | 0.69 | -0.07 | -0.43, 0.26 | 0.68 | -0.05 | 0.80 | -0.03 | -0.40, 0.37 | 0.86 |
|  |  | Δ tCho | 0.08 | 0.68 | 0.08 | -0.24, 0.51 | 0.69 | 0.08 | 0.68 | 0.07 | -0.26, 0.49 | 0.70 |
| Δ VFC | 31 | Baseline tCho | -0.13 | 0.48 | -0.17 | -0.45, 0.12 | 0.26 | -0.18 | 0.32 | -0.22 | -0.52, 0.15 | 0.20 |
|  |  | Δ tCho | 0.27 | 0.14 | 0.21 | -0.21, 0.59 | 0.30 | 0.31 | 0.09 | 0.25 | -0.15, 0.62 | 0.19 |
| Δ SWR | 31 | Baseline tCho | 0.25 | 0.18 | 0.24 | -0.02, 0.44 | **0.04** | 0.30 | 0.10 | 0.32 | 0.02, 0.57 | **0.02** |
|  |  | Δ tCho | -0.05 | 0.77 | -0.09 | -0.34, 0.20 | 0.52 | -0.05 | 0.77 | -0.10 | -0.41, 0.16 | 0.49 |
| Δ Whole brain | 30 | Baseline tCho | 0.14 | 0.48 | 0.09 | -0.37, 0.51 | 0.70 | 0.30 | 0.10 | 0.26 | -0.16, 0.64 | 0.20 |
|  |  | Δ tCho | 0.10 | 0.58 | 0.00 | -0.40, 0.38 | 0.99 | 0.07 | 0.72 | -0.05 | -0.51, 0.39 | 0.81 |
| Δ Caudate | 31 | Baseline tCho | 0.25 | 0.18 | 0.18 | -0.16, 0.60 | 0.35 | 0.41 | **0.02** | 0.34 | 0.04, 0.66 | **0.04** |
|  |  | Δ tCho | 0.10 | 0.58 | 0.06 | -0.25, 0.35 | 0.69 | 0.05 | 0.78 | -0.01 | -0.28, 0.29 | 0.97 |
| Δ White matter | 30 | Baseline tCho | -0.05 | 0.79 | -0.10 | -0.45, 0.30 | 0.60 | 0.15 | 0.43 | 0.09 | -0.24, 0.44 | 0.62 |
|  |  | Δ tCho | 0.34 | 0.07 | 0.28 | -0.05, 0.65 | 0.13 | 0.31 | 0.09 | 0.22 | -0.13, 0.71 | 0.29 |
| Δ Grey matter | 30 | Baseline tCho | -0.04 | 0.83 | -0.08 | -0.46, 0.39 | 0.71 | 0.08 | 0.67 | 0.02 | -0.28, 0.41 | 0.91 |
|  |  | Δ tCho | 0.30 | 0.11 | 0.25 | -0.05, 0.53 | 0.09 | 0.26 | 0.17 | 0.19 | -0.10, 0.53 | 0.22 |

| **MI** | |  | **Age, PVE-adjusted** | | | | | **Age, PVE and**  **CAG-adjusted** | | | | |
| --- | --- | --- | --- | --- | --- | --- | --- | --- | --- | --- | --- | --- |
|  | |  | **Inverse weighted** | | **Bootstrapped** | | | **Inverse weighted** | | **Bootstrapped** | | |
| **Measures** | **n** | **Association with** | **r** | ***p* value** | **r** | **95 % CIs** | ***p* value** | **r** | ***p* value** | **r** | **95 % CIs** | ***p* value** |
| Δ cUHDRS | 31 | Baseline MI | -0.08 | 0.67 | -0.06 | -0.31, 0.19 | 0.62 | 0.01 | 0.97 | 0.04 | -0.24, 0.29 | 0.77 |
|  |  | Δ MI | 0.00 | 0.99 | -0.08 | -0.39, 0.24 | 0.63 | -0.14 | 0.45 | -0.20 | -0.52, 0.11 | 0.21 |
| Δ DBS | 31 | Baseline MI | 0.36 | 0.05 | 0.33 | -0.03, 0.61 | 0.04 | NA | NA | NA | NA | NA |
|  |  | Δ MI | -0.25 | 0.18 | -0.21 | -0.55, 0.29 | 0.30 | NA | NA | NA | NA | NA |
| Δ TFC | 31 | Baseline MI | -0.12 | 0.51 | -0.09 | -0.49, 0.23 | 0.62 | -0.05 | 0.78 | -0.01 | -0.35, 0.33 | 0.94 |
|  |  | Δ MI | 0.25 | 0.18 | 0.06 | -0.48, 0.49 | 0.82 | 0.06 | 0.75 | -0.11 | -0.60, 0.26 | 0.60 |
| Δ TMS | 31 | Baseline MI | -0.00 | 0.99 | -0.04 | -0.37, 0.22 | 0.76 | -0.03 | 0.87 | -0.08 | -0.41, 0.17 | 0.58 |
|  |  | Δ MI | -0.14 | 0.45 | -0.11 | -0.46, 0.15 | 0.47 | -0.07 | 0.69 | -0.07 | -0.56, 0.25 | 0.73 |
| Δ SDMT | 31 | Baseline MI | -0.04 | 0.85 | -0.03 | -0.29, 0.25 | 0.84 | 0.03 | 0.87 | 0.03 | -0.31, 0.31 | 0.82 |
|  |  | Δ MI | -0.05 | 0.80 | -0.06 | -0.39, 0.28 | 0.75 | -0.09 | 0.64 | -0.08 | -0.51, 0.29 | 0.72 |
| Δ SCN | 31 | Baseline MI | -0.23 | 0.22 | -0.23 | -0.56, 0.14 | 0.20 | -0.20 | 0.28 | -0.20 | -0.57, 0.13 | 0.26 |
|  |  | Δ MI | -0.23 | 0.21 | -0.19 | -0.55, 0.11 | 0.25 | -0.29 | 0.11 | -0.23 | -0.58, 0.18 | 0.22 |
| Δ VFC | 31 | Baseline MI | -0.04 | 0.82 | -0.02 | -0.38, 0.34 | 0.93 | -0.10 | 0.59 | -0.08 | -0.42, 0.27 | 0.66 |
|  |  | Δ MI | -0.01 | 0.97 | -0.01 | -0.42, 0.42 | 0.97 | 0.05 | 0.79 | 0.07 | -0.37, 0.49 | 0.74 |
| Δ SWR | 31 | Baseline MI | -0.04 | 0.82 | -0.07 | -0.33, 0.21 | 0.59 | 0.01 | 0.96 | 0.00 | -0.27, 0.26 | 0.98 |
|  |  | Δ MI | -0.31 | 0.09 | -0.29 | -0.58, 0.07 | 0.10 | -0.35 | 0.05 | -0.33 | -0.56, 0.04 | **0.03** |
| Δ Whole brain | 30 | Baseline MI | -0.43 | **0.02** | -0.46 | -0.70, -0.05 | **<0.01** | -0.31 | 0.09 | -0.37 | -0.65, 0.14 | 0.06 |
|  |  | Δ MI | 0.23 | 0.23 | 0.17 | -0.31, 0.49 | 0.41 | 0.06 | 0.74 | 0.03 | -0.35, 0.40 | 0.89 |
| Δ Caudate | 31 | Baseline MI | -0.40 | **0.03** | -0.44 | -0.68, -0.15 | **<0.01** | -0.36 | 0.05 | -0.38 | -0.63, -0.09 | **<0.01** |
|  |  | Δ MI | 0.18 | 0.32 | 0.11 | -0.35, 0.50 | 0.61 | 0.03 | 0.89 | -0.04 | -0.40, 0.32 | 0.85 |
| Δ White matter | 30 | Baseline MI | -0.53 | **<0.01** | -0.53 | -0.75, -0.17 | **<0.01** | -0.46 | **<0.01** | -0.47 | -0.72, -0.08 | **<0.01** |
|  |  | Δ MI | 0.36 | 0.05 | 0.22 | -0.42, 0.66 | 0.41 | 0.21 | 0.27 | 0.07 | -0.48, 0.53 | 0.81 |
| Δ Grey matter | 30 | Baseline MI | -0.44 | **0.02** | -0.43 | -0.65, -0.08 | **<0.01** | -0.40 | **0.02** | -0.40 | -0.64, -0.11 | **<0.01** |
|  |  | Δ MI | 0.32 | 0.08 | 0.23 | -0.11, 0.56 | 0.18 | 0.18 | 0.33 | 0.11 | -0.20, 0.41 | 0.49 |

| **GSH** | |  | **Age, PVE-adjusted** | | | | | **Age, PVE and**  **CAG-adjusted** | | | | |
| --- | --- | --- | --- | --- | --- | --- | --- | --- | --- | --- | --- | --- |
|  | |  | **Inverse weighted** | | **Bootstrapped** | | | **Inverse weighted** | | **Bootstrapped** | | |
| **Measures** | **n** | **Association with** | **r** | ***p* value** | **r** | **95 % CIs** | ***p* value** | **r** | ***p* value** | **r** | **95 % CIs** | ***p* value** |
| Δ cUHDRS | 31 | Baseline GSH | 0.29 | 0.11 | 0.31 | 0.01, 0.57 | **0.03** | 0.30 | 0.10 | 0.31 | 0.03, 0.55 | **0.02** |
|  |  | Δ GSH | -0.03 | 0.87 | -0.07 | -0.42, 0.30 | 0.71 | -0.08 | 0.67 | -0.14 | -0.45, 0.19 | 0.42 |
| Δ DBS | 31 | Baseline GSH | 0.07 | 0.70 | 0.03 | -0.36, 0.33 | 0.85 | NA | NA | NA | NA | NA |
|  |  | Δ GSH | -0.02 | 0.90 | -0.13 | -0.47, 0.23 | 0.49 | NA | NA | NA | NA | NA |
| Δ TFC | 31 | Baseline GSH | 0.12 | 0.51 | 0.15 | -0.16, 0.45 | 0.33 | 0.11 | 0.55 | 0.14 | -0.16, 0.42 | 0.34 |
|  |  | Δ GSH | 0.14 | 0.47 | -0.00 | -0.38, 0.38 | 0.99 | 0.06 | 0.76 | -0.10 | -0.45, 0.24 | 0.57 |
| Δ TMS | 31 | Baseline GSH | -0.08 | 0.66 | -0.12 | -0.36, 0.24 | 0.40 | -0.09 | 0.64 | -0.12 | -0.36, 0.30 | 0.43 |
|  |  | Δ GSH | -0.20 | 0.28 | -0.15 | -0.40, 0.15 | 0.28 | -0.19 | 0.31 | -0.13 | -0.41, 0.16 | 0.35 |
| Δ SDMT | 31 | Baseline GSH | 0.29 | 0.11 | 0.29 | 0.01, 0.60 | 0.06 | 0.29 | 0.11 | 0.28 | -0.02, 0.57 | 0.06 |
|  |  | Δ GSH | -0.14 | 0.45 | -0.14 | -0.48, 0.26 | 0.48 | -0.15 | 0.43 | -0.15 | -0.52, 0.26 | 0.46 |
| Δ SCN | 31 | Baseline GSH | 0.22 | 0.24 | 0.20 | -0.21, 0.53 | 0.31 | 0.22 | 0.23 | 0.19 | -0.24, 0.53 | 0.34 |
|  |  | Δ GSH | -0.30 | 0.10 | -0.20 | -0.49, 0.14 | 0.22 | -0.31 | 0.09 | -0.22 | -0.49, 0.08 | 0.14 |
| Δ VFC | 31 | Baseline GSH | 0.17 | 0.36 | 0.20 | -0.12, 0.52 | 0.22 | 0.20 | 0.29 | 0.22 | -0.11, 0.53 | 0.18 |
|  |  | Δ GSH | -0.03 | 0.87 | -0.06 | -0.41, 0.40 | 0.79 | 0.00 | 0.99 | -0.01 | -0.39, 0.42 | 0.95 |
| Δ SWR | 31 | Baseline GSH | 0.26 | 0.17 | 0.23 | -0.22, 0.52 | 0.22 | 0.26 | 0.16 | 0.21 | -0.27, 0.49 | 0.26 |
|  |  | Δ GSH | -0.26 | 0.15 | -0.15 | -0.45, 0.24 | 0.40 | -0.25 | 0.17 | -0.17 | -0.44, 0.20 | 0.29 |
| Δ Whole brain | 30 | Baseline GSH | 0.43 | **0.02** | 0.41 | 0.04, 0.69 | **0.01** | 0.41 | **0.02** | 0.39 | 0.02, 0.69 | **0.03** |
|  |  | Δ GSH | -0.02 | 0.92 | 0.06 | -0.33, 0.50 | 0.79 | -0.11 | 0.55 | -0.06 | -0.43, 0.37 | 0.78 |
| Δ Caudate | 31 | Baseline GSH | 0.25 | 0.18 | 0.28 | -0.09, 0.49 | 0.12 | 0.25 | 0.18 | 0.21 | -0.15, 0.44 | 0.13 |
|  |  | Δ GSH | 0.06 | 0.75 | 0.13 | -0.23, 0.42 | 0.43 | 0.01 | 0.95 | 0.06 | -0.26, 0.36 | 0.70 |
| Δ White matter | 30 | Baseline GSH | 0.31 | 0.09 | 0.30 | -0.07, 0.62 | 0.09 | 0.34 | 0.07 | 0.30 | -0.05, 0.59 | 0.07 |
|  |  | Δ GSH | 0.12 | 0.53 | 0.13 | -0.31, 0.53 | 0.55 | 0.05 | 0.79 | 0.04 | -0.36, 0.43 | 0.86 |
| Δ Grey matter | 30 | Baseline GSH | 0.17 | 0.38 | 0.16 | -0.17, 0.47 | 0.34 | 0.16 | 0.40 | 0.14 | -0.16, 0.45 | 0.37 |
|  |  | Δ GSH | 0.19 | 0.32 | 0.23 | -0.14, 0.57 | 0.22 | 0.15 | 0.44 | 0.17 | -0.16, 0.50 | 0.31 |

| **GABA** | |  | **Age, PVE-adjusted** | | | | | **Age, PVE and**  **CAG-adjusted** | | | | |
| --- | --- | --- | --- | --- | --- | --- | --- | --- | --- | --- | --- | --- |
|  | |  | **Inverse weighted** | | **Bootstrapped** | | | **Inverse weighted** | | **Bootstrapped** | | |
| **Measures** | **n** | **Association with** | **r** | ***p* value** | **r** | **95 % CIs** | ***p* value** | **r** | ***p* value** | **r** | **95 % CIs** | ***p* value** |
| Δ cUHDRS | 22 | Baseline GABA | 0.02 | 0.93 | -0.18 | -0.57, 0.41 | 0.47 | 0.01 | 0.96 | -0.21 | -0.61, 0.36 | 0.37 |
|  |  | Δ GABA | 0.36 | 0.10 | 0.29 | -0.40, 0.66 | 0.22 | 0.37 | 0.09 | 0.32 | -0.33, 0.63 | 0.14 |
| Δ DBS | 22 | Baseline GABA | -0.08 | 0.72 | -0.14 | -0.59, 0.26 | 0.53 | NA | NA | NA | NA | NA |
|  |  | Δ GABA | 0.00 | 0.99 | 0.07 | -0.48, 0.53 | 0.80 | NA | NA | NA | NA | NA |
| Δ TFC | 22 | Baseline GABA | -0.10 | 0.65 | -0.15 | -0.71, 0.27 | 0.56 | -0.12 | 0.59 | -0.20 | -0.70, 0.22 | 0.43 |
|  |  | Δ GABA | 0.12 | 0.60 | 0.09 | -0.29, 0.43 | 0.64 | 0.15 | 0.52 | 0.12 | -0.20, 0.48 | 0.47 |
| Δ TMS | 22 | Baseline GABA | -0.03 | 0.90 | 0.16 | -0.29, 0.74 | 0.50 | -0.01 | 0.96 | 0.20 | -0.29, 0.71 | 0.41 |
|  |  | Δ GABA | -0.25 | 0.26 | -0.20 | -0.51, 0.32 | 0.32 | -0.25 | 0.26 | -0.21 | -0.55, 0.30 | 0.31 |
| Δ SDMT | 22 | Baseline GABA | -0.16 | 0.48 | -0.27 | -0.51, 0.04 | 0.05 | -0.13 | 0.55 | -0.25 | -0.50, 0.05 | 0.08 |
|  |  | Δ GABA | 0.42 | 0.05 | 0.41 | 0.17, 0.61 | **<0.01** | 0.41 | 0.06 | 0.41 | 0.16, 0.59 | **<0.01** |
| Δ SCN | 22 | Baseline GABA | -0.17 | 0.45 | -0.16 | -0.52, 0.21 | 0.39 | -0.19 | 0.41 | -0.18 | -0.53, 0.18 | 0.34 |
|  |  | Δ GABA | 0.33 | 0.14 | 0.32 | -0.05, 0.60 | 0.05 | 0.32 | 0.15 | 0.33 | -0.00, 0.59 | **0.03** |
| Δ VFC | 22 | Baseline GABA | 0.06 | 0.81 | -0.07 | -0.41, 0.35 | 0.70 | 0.15 | 0.51 | 0.02 | -0.35, 0.46 | 0.93 |
|  |  | Δ GABA | 0.16 | 0.46 | 0.14 | -0.25, 0.44 | 0.43 | 0.15 | 0.51 | 0.12 | -0.23, 0.43 | 0.50 |
| Δ SWR | 22 | Baseline GABA | 0.25 | 0.27 | 0.10 | -0.54, 0.63 | 0.76 | 0.23 | 0.31 | 0.08 | -0.54, 0.63 | 0.78 |
|  |  | Δ GABA | 0.08 | 0.71 | 0.05 | -0.63, 0.52 | 0.88 | 0.07 | 0.75 | 0.05 | -0.63, 0.50 | 0.88 |
| Δ Whole brain | 21 | Baseline GABA | 0.16 | 0.49 | 0.17 | -0.23, 0.42 | 0.28 | 0.11 | 0.63 | 0.14 | -0.13, 0.38 | 0.30 |
|  |  | Δ GABA | -0.07 | 0.75 | -0.09 | -0.40, 0.24 | 0.60 | -0.03 | 0.90 | -0.05 | -0.41, 0.34 | 0.79 |
| Δ Caudate | 22 | Baseline GABA | 0.03 | 0.91 | -0.00 | -0.33, 0.36 | 0.99 | -0.04 | 0.85 | -0.09 | -0.40, 0.27 | 0.61 |
|  |  | Δ GABA | -0.08 | 0.73 | -0.15 | -0.62, 0.28 | 0.51 | -0.08 | 0.72 | -0.15 | -0.60, 0.27 | 0.51 |
| Δ White matter | 22 | Baseline GABA | 0.09 | 0.70 | 0.08 | -0.46, 0.41 | 0.72 | 0.05 | 0.82 | 0.00 | -0.37, 0.32 | 0.98 |
|  |  | Δ GABA | 0.05 | 0.82 | 0.01 | -0.36, 0.30 | 0.96 | 0.08 | 0.71 | 0.04 | -0.37, 0.39 | 0.82 |
| Δ Grey matter | 22 | Baseline GABA | -0.08 | 0.71 | -0.08 | -0.48, 0.29 | 0.68 | -0.12 | 0.60 | -0.12 | -0.44, 0.19 | 0.48 |
|  |  | Δ GABA | 0.10 | 0.67 | 0.05 | -0.32, 0.32 | 0.78 | 0.12 | 0.59 | 0.07 | -0.39, 0.40 | 0.71 |

| **GLX** | |  | **Age, PVE-adjusted** | | | | | **Age, PVE and**  **CAG-adjusted** | | | | |
| --- | --- | --- | --- | --- | --- | --- | --- | --- | --- | --- | --- | --- |
|  | |  | **Inverse weighted** | | **Bootstrapped** | | | **Inverse weighted** | | **Bootstrapped** | | |
| **Measures** | **n** | **Association with** | **r** | ***p* value** | **r** | **95 % CIs** | ***p* value** | **r** | ***p* value** | **r** | **95 % CIs** | ***p* value** |
| Δ cUHDRS | 31 | Baseline GLX | 0.11 | 0.56 | 0.15 | -0.28, 0.55 | 0.49 | 0.08 | 0.68 | 0.10 | -0.40, 0.46 | 0.67 |
|  |  | Δ GLX | 0.17 | 0.36 | 0.16 | -0.33, 0.46 | 0.40 | 0.23 | 0.21 | 0.21 | -0.21, 0.49 | 0.21 |
| Δ DBS | 31 | Baseline GLX | 0.13 | 0.49 | 0.04 | -0.33, 0.40 | 0.85 | NA | NA | NA | NA | NA |
|  |  | Δ GLX | -0.02 | 0.93 | -0.06 | -0.42, 0.33 | 0.76 | NA | NA | NA | NA | NA |
| Δ TFC | 31 | Baseline GLX | 0.18 | 0.33 | 0.23 | -0.05, 0.51 | 0.11 | 0.17 | 0.36 | 0.20 | -0.07, 0.44 | 0.13 |
|  |  | Δ GLX | 0.12 | 0.51 | 0.15 | -0.23, 0.46 | 0.40 | 0.21 | 0.26 | 0.23 | -0.08, 0.52 | 0.13 |
| Δ TMS | 31 | Baseline GLX | -0.14 | 0.44 | -0.18 | -0.44, 0.21 | 0.25 | -0.15 | 0.43 | -0.17 | -0.43, 0.20 | 0.26 |
|  |  | Δ GLX | -0.35 | 0.06 | -0.32 | -0.48, 0.01 | **<0.01** | -0.38 | **0.03** | -0.35 | -0.50, -0.01 | **<0.01** |
| Δ SDMT | 31 | Baseline GLX | -0.01 | 0.96 | 0.08 | -0.33, 0.59 | 0.73 | -0.02 | 0.89 | 0.04 | -0.31, 0.46 | 0.85 |
|  |  | Δ GLX | -0.11 | 0.56 | -0.14 | -0.56, 0.18 | 0.45 | -0.10 | 0.58 | -0.14 | -0.50, 0.20 | 0.43 |
| Δ SCN | 31 | Baseline GLX | -0.18 | 0.34 | -0.12 | -0.57, 0.44 | 0.65 | -0.22 | 0.24 | -0.18 | -0.62, 0.38 | 0.48 |
|  |  | Δ GLX | -0.05 | 0.79 | -0.00 | -0.40, 0.43 | 0.99 | -0.04 | 0.83 | 0.00 | -0.42, 0.41 | 0.98 |
| Δ VFC | 31 | Baseline GLX | -0.13 | 0.48 | -0.03 | -0.40, 0.40 | 0.86 | -0.09 | 0.63 | 0.00 | -0.38, 0.38 | 0.98 |
|  |  | Δ GLX | 0.15 | 0.43 | 0.06 | -0.43, 0.44 | 0.79 | 0.12 | 0.50 | 0.03 | -0.45, 0.42 | 0.87 |
| Δ SWR | 31 | Baseline GLX | -0.05 | 0.77 | -0.07 | -0.60, 0.43 | 0.79 | -0.11 | 0.55 | -0.14 | -0.64, 0.32 | 0.58 |
|  |  | Δ GLX | 0.11 | 0.55 | 0.13 | -0.27, 0.38 | 0.43 | 0.11 | 0.54 | 0.14 | -0.21, 0.40 | 0.37 |
| Δ Whole brain | 30 | Baseline GLX | 0.37 | 0.05 | 0.37 | -0.02, 0.65 | **0.02** | 0.32 | 0.08 | 0.30 | -0.10, 0.62 | 0.10 |
|  |  | Δ GLX | 0.00 | 0.99 | -0.02 | -0.36, 0.32 | 0.91 | -0.03 | 0.87 | -0.03 | -0.38, 0.30 | 0.86 |
| Δ Caudate | 31 | Baseline GLX | 0.15 | 0.41 | 0.17 | -0.18, 0.46 | 0.31 | 0.09 | 0.63 | 0.08 | -0.39, 0.38 | 0.67 |
|  |  | Δ GLX | 0.17 | 0.36 | 0.21 | -0.18, 0.46 | 0.20 | 0.26 | 0.16 | 0.30 | 0.06, 0.47 | **<0.01** |
| Δ White matter | 30 | Baseline GLX | 0.33 | 0.08 | 0.36 | 0.03, 0.62 | **0.02** | 0.28 | 0.14 | 0.28 | -0.03, 0.51 | **0.04** |
|  |  | Δ GLX | 0.20 | 0.28 | 0.22 | -0.10, 0.50 | 0.15 | 0.36 | 0.05 | 0.35 | 0.13, 0.55 | **<0.01** |
| Δ Grey matter | 30 | Baseline GLX | 0.22 | 0.24 | 0.26 | -0.09, 0.54 | 0.10 | 0.19 | 0.32 | 0.22 | -0.07, 0.48 | 0.13 |
|  |  | Δ GLX | 0.29 | 0.13 | 0.33 | 0.04, 0.60 | **0.02** | 0.41 | **0.02** | 0.45 | 0.23, 0.61 | **<0.01** |

Relationships between MRS metabolites and rate of change (Δ) in clinical, cognitive, and volumetric markers were assessed using Pearson’s partial correlation controlling for age and CSF PVE, and age, CSF PVE and CAG repeat length. When analysing DBS, only CSF PVE was adjusted for. Correlation coefficients and 95% confidence intervals were computed using bootstrap testing with 1000 repetitions. A weighted correlation was also conducted, applying an inverse weighting to %SD value. Results displayed are unadjusted for multiplicity. Bold text indicated significance p <0.05. cUHDRS, composite Unified Huntington’s Disease Rating Scale; DBS, Disease Burden Score; PVE, partial volume effect; TFC, Total Functional Capacity; TMS, Total Motor Score; SDMT, Symbol Digit Modalities Test; SCN, Stroop Colour Naming; VFC, Verbal Fluency Categorical; SWR, Stroop Word Reading Test.
